# Fe(OTf)_3_-catalysed Friedel–Crafts reaction of benzenoid arenes with α,β-unsaturated carbonyl compounds: easy access to 1,1-diarylalkanes

**DOI:** 10.1098/rsos.170748

**Published:** 2017-10-25

**Authors:** Aditya Bhattacharya, Pushpendra Mani Shukla, Biswajit Maji

**Affiliations:** Department of Chemistry, Indira Gandhi National Tribal University, Amarkantak, Madhya Pradesh 484887, India

**Keywords:** Friedel–Crafts alkylation, 1,1-diarylalkane, iron triflate, β,β-diaryl carbonyl compounds

## Abstract

A simple and efficient method for the synthesis of 1,1-diarylalkanes via the Friedel–Crafts-type alkylation reaction of electron-rich arenes with cinnamic acid ester derivatives or chalcones is reported. Iron triflate has been found to be the best catalyst for the Friedel–Crafts-type alkylation reaction with α,β-unsaturated carbonyl compounds. This reaction afforded β,β-diaryl carbonyl compounds in good yields (65–93%) and with excellent regioselectivities. Remarkably, this method is also compatible with a variety of indoles to provide 3-indolyl-aryl carbonyl compounds in excellent yields. Great efforts have been made to deduce a plausible reaction mechanism based on isotopic labelling experiments.

## 1. Introduction

**Figure 1.**
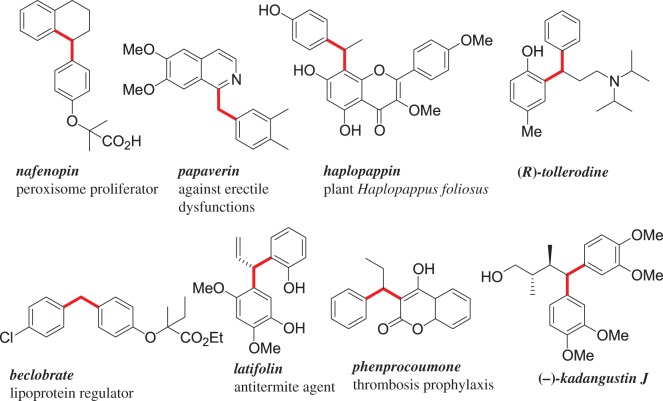
Representative examples of 1,1-diarylalkanes with biological activities.

Friedel–Crafts alkylated compounds are of huge importance for the chemical industry as pharmaceuticals, agrochemicals and fine chemicals [1–13]. Especially, the synthesis of 1,1-diarylalkane is highly significant as the core structure of 1,1-diarylalkane is found in many important molecules having biological activities (figure 1). For the preparation of 1,1-diarylalkanes, commonly used alkylating agents are π-activated alcohols, acetates, phosphates and halides in the presence of catalytic amount of Lewis acids or Brönsted acids [14–23]. A significant drawback of large-scale chemical processes is that a vast amount of waste by-products is produced, and it is still a challenging task in standard protocols to free the by-products from arylated products. Three-member cyclic and reactive intermediates, such as epoxides [24–32], aziridines [33–47] and halonium ions [48–53], or other unstable intermediates [54–58] derived *in situ* from alkenes have also been found as potential electrophiles to afford functionalized Friedel–Crafts alkylated products. Another important strategy to afford 1,1-diarylalkanes is Friedel–Crafts-type hydroarylation reaction with olefins. The greatest advantage of these hydroarylation methods is 100% atom-efficient product formations. The Friedel–Crafts-type hydroarylation reaction with olefins, particularly with styrenes has been realized with AlCl_3_ [59,60], BiCl_3_ [61], Bi(OTf)_3_ [62], Ca(NTf_2_)_2_/Bu_4_NPF_6_ [63], FeCl_3_ [64], Au(I) or Au(III)-complexes [65,66], TMSCl/ZnBr_2_ [67], Ru(III) salt [68], Pt complexes [69,70], graphene oxide [71], resins [72], zeolites [73], I_2_ [74] and Bronsted acids [75,76]. Hydroarylation reaction with alkynes is also well investigated [77–82]. The Friedel–Crafts-type alkylation reaction with a variety of Michael acceptors is also extensively investigated, although these reactions are mostly limited to heteroarenes such as indoles, pyrroles and thiophenes [83–92]. By contrast, the intermolecular Friedel–Crafts-type reactions of benzenoid arenes with electron-deficient alkenes such as α,β-unsaturated cinnamic acid ester derivatives and chalcones as Michael acceptors are not recognized as substrates. Therefore, keeping in mind the importance of diarylalkanes (figure 1), it is obvious that there is an urgent need for the development of a sustainable, atom-economical and operationally simple approach.

## 2. Model

**Scheme 1.**
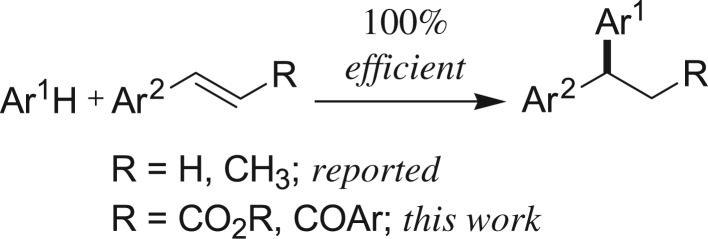
1,1-Diarylalkane synthesis.

Recently, it has been well accepted that iron metal salts (Fe is one of the most abundant metals in the earth's crust, approx. 4.7 wt %) could fulfil a few points of the principles of green chemistry, as iron salt is cheap, easily available, non-toxic and environmentally benign. As a result, a series of novel organic transformations including oxidations, reductions, cross-coupling reactions and hydroarylation reactions have also been developed employing iron salts as Lewis acid [93–96]. As a consequence, we envisaged that the iron salt as Lewis acid could be used in Friedel–Crafts-type alkylation to access 1,1-diarylalkanes (scheme 1).

We, herewith, report an environmentally benign iron triflate-catalysed Friedel–Crafts-type alkylation reaction of electron-rich benzenoid arenes with electron-deficient alkenes such as α,β-unsaturated cinnamic acid ester and chalcone derivatives (scheme 1). Moreover, this reaction provides β,β-diaryl carbonyl compounds; these are interestingly found in many biologically active compounds and pharmaceuticals (figure 1).

## 3. Result and discussion

**Figure 2.**
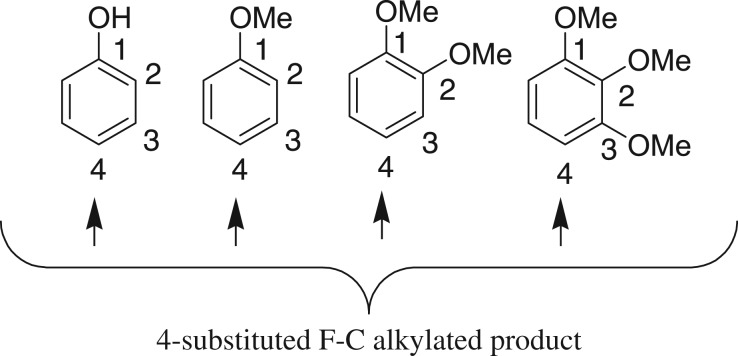
Regioisomers of arene.

For optimization, 3,4-dimethoxy methylcinnamate and 1,2-dimethoxybenzene were selected as model substrates. Initially, the model reaction was conducted in the presence of various commonly available iron metal salts. The FeCl_3_ (anhydrous) in 10 mol%-catalysed hydroarylation reaction afforded the desired compound **3a** in 43% yield along with some uncharacterized by-products when the reaction was carried out in 1,2-dichloroethane solvent at 85°C (table 1, entry 1). As with FeCl_3_ (anhydrous), a similar yield (41%) was obtained when FeCl_3_·6H_2_O was used for the same set of reaction (entry 2). FeBr_3_ (10 mol%) was also tested for this reaction but no improvement in yield (28%) was observed (entry 3). Other iron salts such as Fe_2_O_3_, Fe_2_(SO_4_)_3_, Fe(NO_3_)_2_ 6H_2_O and Fe(OTf)_2_ were completely inactive for this catalytic method (entries 4 and 5). Iron (III) triflate in 10 mol% has been found to be the best for this catalytic process and yielded 78% of **3a** (entry 6). Cu(OTf)_2_ as Lewis acid also furnished the desired product **3a**, albeit in a moderate yield, 51% (entry 7). Other metals such as Zn and Co metals as Lewis acid are completely inactive for this reaction (entries 8 and 9). Bi(OTf)_3_ also furnished the desired compound **3a** in 61% yield (entry 10). Other solvents like nitromethane, *tert*-butanol, dimethoxy ethane and toluene were also examined, but no tested solvent was found suitable for this reaction (entries 11–13). The yield of the Friedel–Crafts alkylated product **3a** was reduced to 47%, while the catalyst loading reduced to 5 mol% (entry 17). Thus, when a suspended solution of 3, 4-dimethoxy methylcinnamate (**1a**) and 1,2-dimethoxybenzene (**2a**) was added to iron (III) triflate (10 mol%) in 1,2-dichloroethane at 85°C, the hydroarylated product **3a** was obtained in 78% yield with excellent regioselectivity. ^1^H-NMR analysis of the crude reaction mixture revealed that no trace amount of other arene or alkene regioisomer was formed. Identifying Fe(OTf)_3_ as a suitable Lewis acid to this catalytic reaction, we began to explore the substrates’ scope (table 2). A variety of electron-rich cinnamate esters and chalcone substrates successfully afforded the desired Friedel–Crafts alkylated products in good to excellent yields. A wide range of electron-rich benzenoid arene compounds yielded Friedel–Crafts alkylated compounds, when the reactions were performed with 3,4-dimethoxy cinnamate ester (table 2; entries 1–4). 4-Methoxy-substituted cinnamate ester also gave the Friedel–Crafts alkylated products in good yields on treatment with electron-donating substituents containing arene compounds (table 2; entries 5–7). More importantly, an arene nucleophile such as phenol was also well tolerated in this catalytic method to afford **3f** in 66% yield (entry 6). 4-Methyl-substituted cinnamate ester also afforded the desired β,β-diaryl carbonyl compound **3h** in 65% yield, when the reactions were performed with electron-rich arene 1,3,5-trimethoxybenzene (entry 8). However, not even a single product was detected from the ^1^H NMR of the crude reaction mixture when the reaction was conducted between methylcinnamate and 1,3,5-trimethoxybenzene (table 2; entry 14). Next, we have extended the scope of this reaction with other α,β-unsaturated carbonyl compounds. We were pleased to find that the iron triflate-catalysed Friedel–Crafts-type alkylation reaction was also applicable for chalcones, and the results are shown in table 2 (entries 9–13). Electron-donating group-substituted arenes were tested with chalcone derivatives such as 3-(3,4-dimethoxy-phenyl)-1-phenyl-propenone and 3-(4-methoxy-phenyl)-1-phenyl-propenone, and they furnished the desired compounds in good to excellent yields (65–86%) (table 2; entries 8–11). Chalcone substrates with methyl or methoxy substituents at the opposite side of the aryl part also participated in this catalytic reaction and afforded **3l** and **3m** with good yields of 86% and 71%, respectively (entries 12–13). Iron triflate-catalysed Friedel–Crafts-type alkylation reaction is limited to electron-rich cinnamate and chalcone derivatives, and it exclusively produced the regioisomer β,β-diaryl carbonyl compounds as arenes preferentially attack the benzylic position. No other regioisomers were detected by ^1^H NMR of the crude reaction mixture. There is also a possibility for the formation of arene regioisomers. For anisole, 1,2-dimethoxybenzene, phenol produced exclusively *para*-alkylated products. By contrast, 1,2,3-trimethoxybenzene produced exclusively 4-substituted product. It seems the electronic effect predominates over the steric factor, i.e. C4 is activated by the +R effect of C1-OMe and C3-OMe, whereas C5-position is *para*-only with C2-OMe (figure 2).

**Table 1.** Optimization study^a^ 
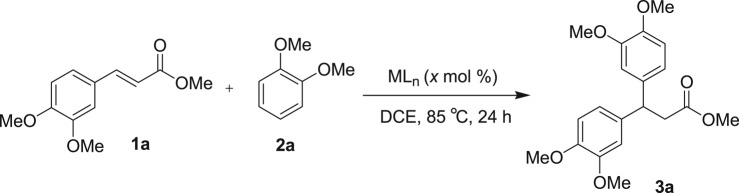

entry	Lewis acid (*x* mol%)	solvent	yield (%) of **3a**^b^
1	FeCl_3_ anhydrous (10)	DCE	43
2	FeCl_3_, 6H_2_O (10)	DCE	41
3	FeBr_3_ (10)	DCE	28
4	Fe_2_O_3_ or Fe_2_(SO4)_3_ or Fe(NO_3_)_2_, 6H_2_O (10)	DCE	0
5	Fe(OTf)_2_ (10)	DCE	0
6	Fe(OTf)_3_ (10)	DCE	78
7	Cu(OTf)_2_ (10)	DCE	51
8	Zn(OTf)_2_ (10)	DCE	0
9	Co(ClO_4_)_2_·6H_2_O or Co(NO_3_)_2_·6H_2_O (10)	DCE	0
10^c^	Bi(OTf)_3_ (10)	DCE	61
11	Fe(OTf)_3_ (10)	CH_3_NO_2_	0
12	Fe(OTf)_3_ (10)	Toluene	0
13	Fe(OTf)_3_ (10)	DME	16
14^d^	Fe(OTf)_3_ (10)	DCE	21
15^e^	Fe(OTf)_3_ (10)	DCE	73
16^f^	Fe(OTf)_3_ (10)	DCE	58
17	Fe(OTf)_3_ (05)	DCE	47

^a^Reaction conditions: alkene **1a** (1.0 equiv, 0.2 mmol), arene **2a** (1.2 equiv) and iron catalyst (10 mol%) are heated to 85°C in solvent (1.0 ml).

^b^Isolated yield after column chromatography.

^c^Bi(OTf)_3_-catalysed same set of reactions in completely anhydrous DCE solvent afforded no product (0% yield).

^d^Reaction was run at 50°C.

^e^1,2-Dimethoxybenzene was used at 5.0 equiv.

^f^Reaction was run at 100°C. DCE = 1,2-dichloroethane.

**Table 2.** Fe(OTf)_3_-catalysed hydroarylation reaction with α,β-unsaturated carbonyl compounds^a^
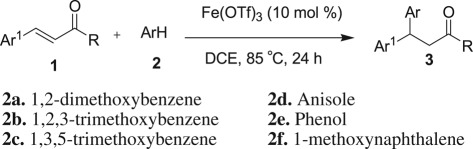

ester or chalcone **1**				
entry	Ar^1^	R	ArH **2**	product **3**, yield (%)^b^
1	3,4-OMeC_6_H_3_	OMe	**2a**	**3a**, 78
2	3,4-OMeC_6_H_3_	OMe	**2b**	**3b**, 73
3	3,4-OMeC_6_H_3_	OMe	**2d**	**3c**, 68
4	3,4-OMeC_6_H_3_	OMe	**2f**	**3d**, 83
5	4-OMeC_6_H_4_	OMe	**2b**	**3e**, 71
6	4-OMeC_6_H_4_	OMe	**2e**	**3f**, 66
7	4-OMeC_6_H_4_	OMe	**2f**	**3g**, 77
8	4-MeC_6_H_4_	OMe	**2c**	**3h**, 65
9	3,4-OMeC_6_H_3_	Ph	**2c**	**3i**, 86
10	4-OMeC_6_H_4_	Ph	**2c**	**3j**, 75
11	4-OMeC_6_H_4_	Ph	**2b**	**3k**, 74
12	4-OMeC_6_H_4_	4-MeC_6_H_4_	**2c**	**3l**, 86
13	Ph	4-OMeC_6_H_4_	**2c**	**3m**, 71
14	Ph	OMe	**2c**	**3n**, 0

^a^Reaction conditions: alkene (1.0 equiv), arene (1.2 equiv) and catalyst Fe(OTf)_3_ (10 mol%) are heated at 85°C in DCE.

^b^Isolated yield after column chromatography.

**Scheme 2.**
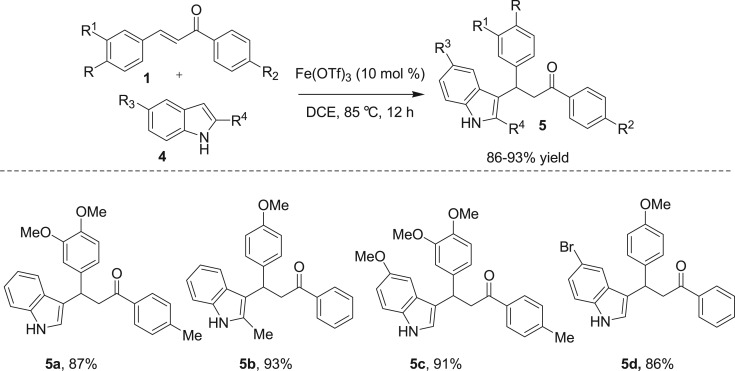
Reaction condition of Friedel–Crafts alkylation reaction of indoles with chalcones: chalcone (1.0 equiv), indole **4** (1.2 equiv) and catalyst Fe(OTf)_3_ (10 mol%) are heated to 85°C in DCE for 12 h. Isolated yield after column chromatography.

Iron (III) triflate was also tested for Friedel–Crafts alkylation reactions between heteroarenes such as a variety of indoles **4** and chalcone derivatives **1** (scheme 2). Successfully, all the reactions yielded the desired 3-indolyl-aryl-substituted carbonyl compounds **5** in good to excellent yields (86–93%) and with excellent regioselectivities.

### 3.1. Mechanism investigation

**Scheme 3.**
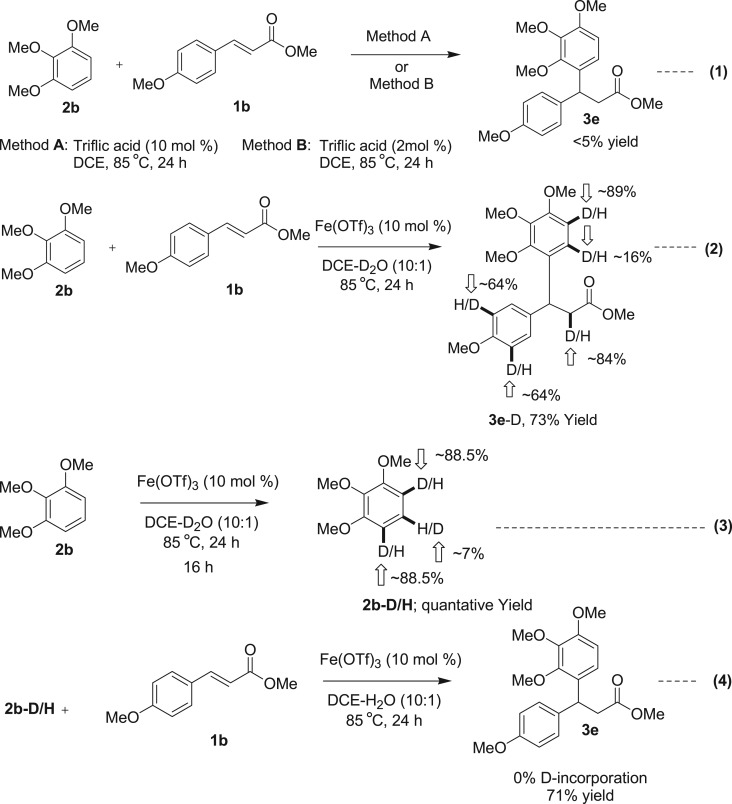
Control experiments to give insight on the reaction mechanism.

**Scheme 4.**
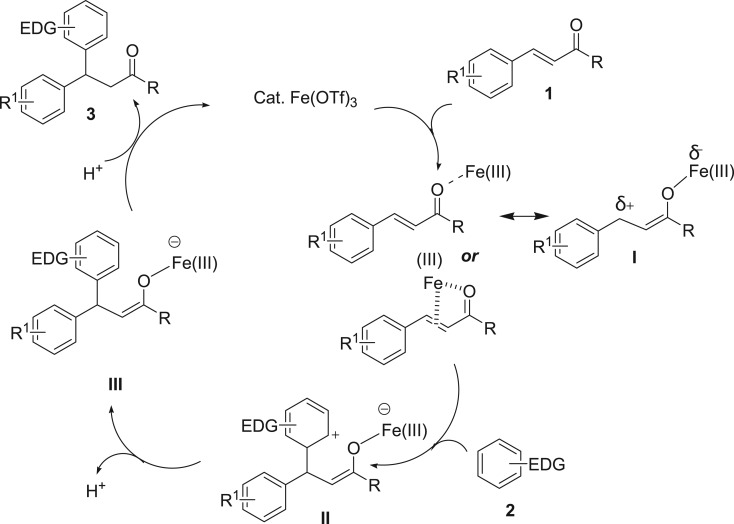
A plausible reaction mechanism.

To gain insight into the reaction mechanism, a series of control reactions including isotopic labelling experiments were performed. First, 4-methoxy methylcinnamate ester was reacted with 1,2,3-trimethoxybenzene in super dry 1,2-dichloroethane (in the presence of molecular sieves 4 Å); the desired compound **3e** was formed in 11% yield under the optimized condition after quenching with H_2_O. Removal of molecular sieves and introduction of wet dichloroethane solvents (without drying the solvent) provided the desired Friedel–Crafts alkylated product **3e** in 72% yield. Therefore, it can be concluded that the concentration of water in the reaction medium is the detrimental factor to obtaining good yields of the product. Next, control experiments using triflic acid, at both catalyst loading (10 mol%) and significantly lower loading (2 mol%) separately, were conducted to examine whether the complex acid forms or not during progress of the reaction (scheme 3; equation (1)). A trace amount of the desired compound **3e** was observed in both the experiments, and the rest of the starting materials were recovered in each case. This experiment clearly suggests the significance of iron (III) triflate as Lewis acid in this catalytic method. Furthermore, to understand a clear mechanism, deuterium labelling experiments were performed in the presence of D_2_O and using deuterated arene separately under the optimized reaction condition. The reaction of 1,2,3-trimethoxybenzene **2b** with 4-methoxy methylcinnamate **1b,** in the presence of D_2_O as co-solvent to ClCH_2_CH_2_Cl, provided **3e-**D in 73% yield with exclusive (approx. 84%) *d*-incorporation at α-carbon of the carbonyl centre. In addition, a significant amount of *d*-incorporations were observed at the arene part of the desired product **3e** (scheme 3; equation (2)). This observation may be attributed to *d*-incorporation taking place at the starting materials first, prior to attack of the alkene or after product formation. Further to verify, arene **2b** was reacted in the presence of D_2_O under the optimized reaction condition and, delightfully, exclusive *d*-incorporation (88.5%) at the C4-H and C6-H of arene **2b**-D/H and 7% *d*-incorporation at the C5-H (scheme 3; equation (3)) were observed. Next, another experiment was carried out with arene **2b**-D/H and alkene **1b** in the presence of ClCH_2_CH_2_Cl–H_2_O (10 : 1). Hydroarylated product **3e** was isolated in 71% yield. Interestingly, no deuterium scrambled either at the α-carbon of the carbonyl centre or at the arene part in **3e** (scheme 3, equation (4)). The isotopic labelling experiment clearly indicates that water plays a significant role in this catalytic reaction and a rapid D/H exchange reaction predominantly occurs in the presence of iron (III) triflate as Lewis acid [97]. Although the mechanism of the present reaction has not been fully established, on the basis of control reactions and isotopic labelling experiments, a Friedel–Crafts-type alkylation reaction is proposed (scheme 4) [98,99]. The reaction would be initiated by activation of the carbonyl group of the starting alkene by iron catalyst and, hence, a partial positive charge at the benzylic centre is developed as intermediate **I**. Alternatively, the iron catalyst may also coordinate with both C=C double bond and the carbonyl group of ester or chalcone derivatives and enhance the reactivity of the benzylic carbon centre. Next, activated arene π-nucleophile attacks the benzylic centre to generate the intermediate **II**. Finally, aromatization of the intermediate **II** and subsequent protonation would provide the desired Friedel–Crafts alkylated product **3**.

## 4. Conclusion

In conclusion, the present methodology demonstrates the iron triflate-catalysed Friedel–Crafts-type alkylation reaction between α,β-unsaturated carbonyl compounds and electron-rich arene components. This reaction provides exclusively β,β-diaryl carbonyl compounds in good to excellent yields. Usually, electro-rich activated arenes gave the desired Friedel–Crafts alkylated products in good to excellent yields in this catalytic method. Heteroarenes such as a variety of indoles also gave the desired 3-indolyl-aryl carbonyl compounds in excellent yields and with excellent regioselectivities. A series of control reactions including isotopic labelling experiments helps to deduce a plausible reaction mechanism. This successful catalytic method encouraged us to accomplish the formal synthesis of a 1,1-diaryl motif containing molecules such as nafenopin, latifolin and tollerodine, which is ongoing in our laboratory.

## Supplementary Material

Electronic Supplementary Information

## Data accessibility

The datasets supporting this article have been uploaded as part of the electronic supplementary material.
