# Supplementary material for: Fe(OTf)3-catalysed Friedel–Crafts reaction of benzenoid arenes with α,β-unsaturated carbonyl compounds: easy access to 1,1-diarylalkanes
Source: R Soc Open Sci. 2017 Oct 25;4(10):170748. doi: 10.1098/rsos.170748 (PMC5666261; doi:10.1098/rsos.170748)
Supplement: Electronic Supplementary Information [file rsos170748supp1.pdf]

Electronic Supporting Information

$Fe(OTf)_3$ Catalyzed Friedel-Crafts Reaction of Benzenoid Arenes with α, β-Unsaturated Carbonyl Compounds: Easy Access to 1,1-Diarylalkanes

Aditya Bhattacharya, Pushpendra mani Shukla and Biswajit Maji*

Serial No	Particulars	Page No
01	General Information	**2**
02	General Reaction Procedure	**2**
03	Spectral Data of all the Friedel-Crafts Alkylated Products **3a-3m**	**3-10**
04	Spectral Data of all the Friedel-Crafts Alkylated Products **5a-5d**	**10-13**
05	Copy of ^{1}H NMR and ^{13}C Spectra of Friedel-Crafts Alkylated Products **3a-3m**	**13-42**
06	Copy of ^{1}H NMR and ^{13}C Spectra of Friedel-Crafts Alkylated Products **5a-5d**	**43-49**
07	Mechanism Investigation	**50-57**

Department of Chemistry
Indira Gandhi National Tribal University
Amarkantak, Madhya Pradesh-484887
India
Email: biswajit.maji@igntu.ac.in

General Information

All reactions were conducted using oven-dried glassware under an atmosphere of Argon (Ar). Commercial grade reagents were used without further purification. Solvents were dried and distilled following usual protocols. 1,2-Dichloroethylene was used in substrates scope (Bottle solvent) directly as such received from Himedia, India. Flash chromatography was carried out using Spectrochem Silica gel (230-400 mesh) purchased from Spectrochem, India. TLC was performed on aluminum-backed plates coated with Silica gel 60 with F_{254} indicator (Merck).

The ^{1}H NMR spectra were measured with Bruker-400 (200 MHz) and ^{13}C NMR spectra were measured with Bruker-200 (50 MHz) using $CDCl_3$. ^{1}H NMR chemical shifts are expressed in parts per million (δ) downfield to $CHCl_3$ (δ = 7.26), ^{13}C NMR chemical shifts are expressed in parts per million (δ) relative to the central $CDCl_3$ resonance (δ = 77.0. Coupling constants in ^{1}H NMR are in Hz. The following abbreviations classify the multiplicity: s = singlet, d = doublet, t = triplet, m = multiplet or unresolved, dd = doublet of doublet. HR-MS (ESI) spectra were recorded on a Waters Q-Tof premier™ mass spectrometer.

General Procedure for the Friedel-Crafts alkylation reaction with cinnamate esters and chalcones:

To a well stirred solution of cinnamate esters or chalcones **1** (1.0 equiv, 0.4 mmol) and arene **2** or **4** (1.2 equiv) in dichloroethane (2.0 mL) was added iron (III) triflate Lewis acid (10 mol %) under argon atmosphere. Next the reaction mixture is allowed to heat at 85 °C for 24 h. Progress of the reaction was monitored by TLC. On completion of the reaction, the reaction mixture was concentrated under reduced pressure and subjected to put directly in the column chromatography for the purification by using ethyl acetate and petroleum ether (60-80 °C) as eluent.

Representative procedure for the preparation of Friedel-Crafts alkylated product 3a: Under an argon atmosphere, iron (III) triflate (0.1 equiv, 22.6 mg, 0.045 mmol) was added to a solution of 3-(3,4-dimethoxy-phenyl)-acrylic acid methyl ester (1.0 equiv, 100 mg, 0.450 mmol) and 1,2-dimethoxybenzene (1.2 equiv, 75 mg, 0.540 mmol) in dichloroethane (2.5 ml). After stirring at room temperature for 5 minutes, the reaction mixture was slowly heated to 85 °C and continued for 24 hours. After 24 hours, the mixture was concentrated under reduced pressure and

purified under flash chromatography eluted with EtOAc: Petroleum ether (60-80 °C) = 4:1 to afford white solid **3a** (126 mg) in 78%.

<u>Spectral Data of all the Friedel-Crafts Alkylated Products 3a-3m</u>

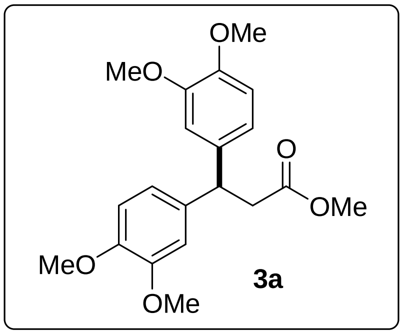

3,3-Bis-(3,4-dimethoxy-phenyl)-propionic acid methyl ester. Yield = 78%, Column purified with EtOAc: Pet Ether = 1:4, White amorphous solid, mp 122-123 °C.

^{1}H NMR ($CDCl_3$, 400 MHz): δ 6.98 (d, *J* = 8.0 Hz, 1H), 6.80-6.77 (m, 3H), 6.44-6.39 (m, 2H), 4.79 (t, *J* = 8.8 Hz, 1H), 3.828 (s, 3H), 3.823 (s, 3H), 3.77 (s, 3H), 3.76 (s, 3H), 3.58 (s, 3H), 2.99 (dd, *J* = 16.0, 7.2 Hz, 2H).

^{13}C-NMR ($CDCl_3$, 100 MHz): δ 172.7, 159.4, 157.7, 148.6, 147.3, 136.1, 128.1, 124.7, 119.4, 111.6, 110.9, 104.0, 98.8, 55.8, 55.78, 55.4, 55.2, 51.5, 39.8, 39.6.

HRMS (EI) calcd for $C_{20}H_{24}O_6$, 361.1651 *m/z* $(M+H)^+$; Found, 361.1654 *m/z*.

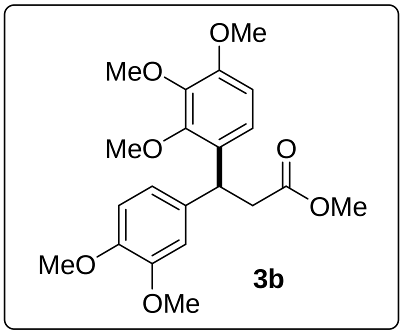

3-(3,4-Dimethoxy-phenyl)-3-(2,3,4-trimethoxy-phenyl)-propionic acid methyl ester. Yield = 73%, Column purified with EtOAc: Pet Ether = 1:4, White amorphous solid, mp 131-132 °C.

¹H NMR ($CDCl_3$, 400 MHz): δ 6.85 (d, *J* = 8.4 Hz, 1H), 6.77-6.75 (m, 3H), 6.60 (d, *J* = 8.4 Hz, 1H), 4.76 (t, *J* = 8.0 Hz, 1H), 3.82 (s, 6H), 3.81 (s, 6H), 3.68 (s, 3H), 3.57 (s, 3H), 2.98 (d, *J* = 8.0 Hz, 2H).

¹³C-NMR ($CDCl_3$, 100 MHz): δ 172.4, 152.4, 151.5, 148.7, 147.4, 142.4, 136.5, 129.7, 121.6, 119.3, 111.4, 111.0, 106.9, 60.7, 60.6, 55.8 (3C), 51.6, 40.2, 40.1.

HRMS (EI) calcd for $C_{20}H_{26}O_7$, 391.1757 *m/z* $(M+H)^+$; Found, 391.1759 *m/z*.

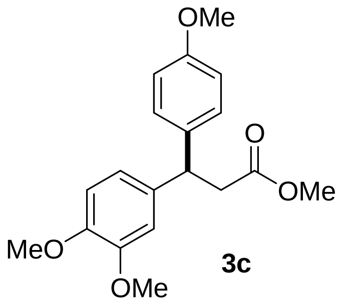

3c

3-(3,4-Dimethoxy-phenyl)-3-(4-methoxy-phenyl)-propionic acid methyl ester. Yield = 68%, Column purified with EtOAc: Pet Ether = 1:5, Gummy liquid.

¹H NMR ($CDCl_3$, 400 MHz): δ 7.13 (d, *J* = 8.4 Hz, 2H), 6.81 (d, *J* = 8.4 Hz, 2H), 6.80-6.78 (m, 2H), 6.71 (s, 1H), 4.45 (t, *J* = 7.6 Hz, 1H), 3.83 (s, 3H), 3.81 (s, 3H), 3.76 (s, 3H), 3.58 (s, 3H), 2.99 (d, *J* = 8.0 Hz, 2H).

¹³C-NMR ($CDCl_3$, 100 MHz): δ 172.5, 158.2, 148.9, 147.7, 136.5, 135.9, 128.6 (2C), 119.3, 114.0 (2C), 111.3, 111.2, 55.94, 55.92, 55.3, 51.8, 45.9, 41.1.

HRMS (EI) calcd for $C_{19}H_{22}O_5$, 331.1546 *m/z* $(M+H)^+$; Found, 331.1551 *m/z*.

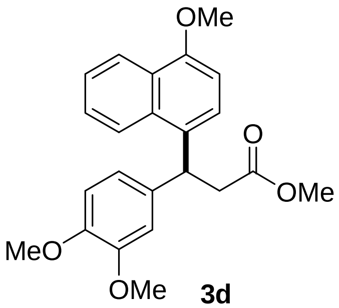

3d

3-(3,4-Dimethoxy-phenyl)-3-(4-methoxy-naphthalen-1-yl)-propionic acid methyl ester. Yield = 83%, Column purified with EtOAc: Pet Ether = 1:4, White amorphous solid, mp 134-136 °C.

^{1}H NMR ($CDCl_3$, 400 MHz): δ 8.30 (d, *J* = 8.0 Hz, 1H), 8.07 (d, *J* = 8.0 Hz, 1H), 7.50-7.44 (m, 2H), 7.25 (d, *J* = 8.0 Hz, 1H), 6.84-6.74 (m, 4H), 5.24 (t, *J* = 8.0 Hz, 1H), 3.98 (s, 3H), 3.84 (s, 3H), 3.80 (s, 3H), 3.61 (s, 3H), 3.12 (dd, *J* = 16.0, 7.2 Hz, 2H).

^{13}C-NMR ($CDCl_3$, 100 MHz): δ 172.5, 154.6, 148.9, 147.6, 136.8, 132.3, 131.0, 126.7, 126.4, 125.5, 124.0, 123.5, 122.6, 119.6, 111.4, 111.1, 103.0, 55.83, 55.80, 55.43, 51.7, 41.9, 41.3.

HRMS (EI) calcd for $C_{23}H_{24}O_5$, 381.1702 *m/z* $(M+H)^+$; Found, 381.1705 *m/z*.

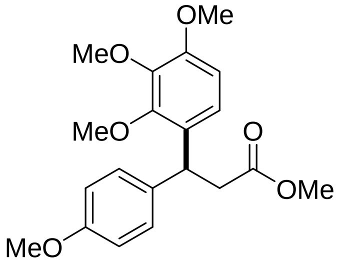

3e

3-(4-Methoxy-phenyl)-3-(2,3,4-trimethoxy-phenyl)-propionic acid methyl ester. Yield = 71%, Column purified with EtOAc: Pet Ether = 1:6, Colorless crystalline solid (Recrystallised from EA-Petroleum ether mixture), mp 127°C.

^{1}H NMR ($CDCl_3$, 400 MHz): δ 7.14 (d, *J* = 8.8 Hz, 2H), 6.87 (d, *J* = 8.8 Hz, 1H), 6.80 (d, *J* = 8.8 Hz, 2H), 6.61 (d, *J* = 8.8 Hz, 1H), 4.77 (t, *J* = 8.0 Hz, 1H), 3.84 (s, 3H), 3.83 (s, 3H), 3.75 (s, 3H), 3.67 (s, 3H), 3.58 (s, 3H), 2.98 (d, *J* = 7.6 Hz, 2H).

^{13}C-NMR (CDCl$_3$, 100 MHz): δ 172.7, 158.1, 152.6, 151.7, 142.6, 136.1, 130.6, 128.8 (2C), 121.7, 113.9 (2C), 107.0, 60.83, 60.79, 56.0, 55.3, 51.8, 40.4, 39.9.

HRMS (EI) calcd for $C_{20}H_{24}O_6$, 361.1651 *m/z* $(M+H)^+$; Found, 361.1653 *m/z*.

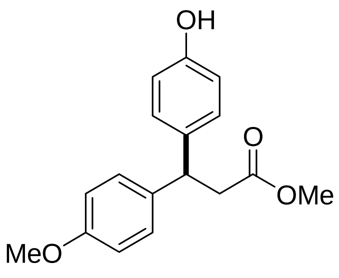

3f

3-(4-Hydroxy-phenyl)-3-(4-methoxy-phenyl)-propionic acid methyl ester. Yield = 66%, Column purified with EtOAc: Pet Ether = 1:6, Colorless cubic crystal (Recrystallised from MeOH), mp 130 °C.

^{1}H NMR (CDCl$_3$, 400 MHz): δ 7.12 (d, *J* = 8.2 Hz, 2H), 7.05 (d, *J* = 8.2 Hz, 2H), 6.81 (d, *J* = 8.4 Hz, 2H), 6.76 (d, *J* = 8.2 Hz, 2H), 6.02 (s, 1H), 4.44 (t, *J* = 8.0 Hz, 1H), 3.73 (s, 3H), 3.59 (s, 3H), 3.01 (d, *J* = 8.4 Hz, 2H).

^{13}C-NMR (CDCl$_3$, 100 MHz): δ 172.9, 157.9, 154.3, 135.9, 135.5, 128.56 (2C), 128.4 (2C), 115.35 (2C), 113.8 (2C), 55.16, 51.8, 45.3, 41.0.

HRMS (EI) calcd for $C_{17}H_{18}O_4$, 287.1283 *m/z* $(M+H)^+$; Found, 287.1283 *m/z*.

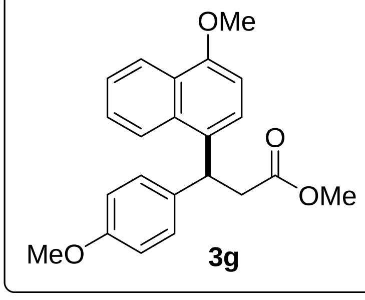

3g

3-(4-Methoxy-naphthalen-1-yl)-3-(4-methoxy-phenyl)-propionic acid methyl ester. Yield = 77%, Column purified with EtOAc: Pet Ether = 1:5, Gummy liquid.

^{1}H NMR (CDCl$_3$, 400 MHz): δ 8.27 (d, *J* = 8.4 Hz, 1H), 8.03 (d, *J* = 8.0 Hz, 1H), 7.50-7.38 (m, 2H), 7.25 (d, *J* = 8.0 Hz, 1H), 7.16 (d, *J* = 8.8 Hz, 2H), 6.77 (d, *J* = 8.8 Hz, 2H), 6.74 (d, *J* = 8.8 Hz, 1H), 5.22 (t, *J* = 8.0 Hz, 1H), 3.94 (s, 3H), 3.70 (s, 3H), 3.57 (s, 3H), 3.17-3.03 (m, 2H).

^{13}C-NMR (CDCl$_3$, 100 MHz): δ 172.7, 158.2, 154.7, 136.0, 132.5, 131.26, 128.9 (2C), 126.8, 126.3, 125.0, 124.1, 123.7, 122.7, 114.1 (2C), 103.2, 55.6, 55.3, 51.8, 41.67, 41.57.

HRMS (EI) calcd for $C_{22}H_{22}O_4$, 351.1596 *m/z* $(M+H)^+$; Found, 351.1597 *m/z*.

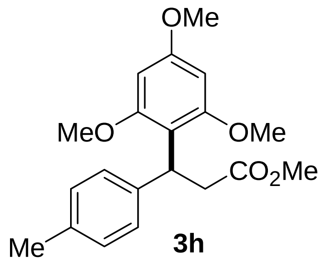

3h

3-*p*-Tolyl-3-(2,4,6-trimethoxy-phenyl)-propionic acid methyl ester. Yield = 65%, Column purified with EtOAc: Pet Ether = 1:7, Gummy liquid.

^{1}H NMR (CDCl$_3$, 400 MHz): δ 7.20 (d, *J* = 8.0 Hz, 2H), 7.02 (d, *J* = 8.0 Hz, 2H), 6.10 (s, 2H), 5.13 (t, *J* = 8.0 Hz, 1H), 3.77 (s, 3H), 3.76 (s, 6H), 3.58 (s, 3H), 3.30 (dd, *J* = 15.6, 7.6 Hz, 1H), 3.15 (dd, *J* = 15.6, 7.6 Hz, 1H), 2.27 (s, 3H).

^{13}C-NMR (CDCl$_3$, 100 MHz): δ 173.7, 159.7, 159.0 (2C), 141.0, 134.9, 128.5 (2C), 127.4 (2C), 112.8, 91.2 (2C), 55.76 (2C), 55.2, 51.4, 37.7, 35.4, 21.0.

HRMS (EI) calcd for $C_{20}H_{24}O_5$, 345.1702 *m/z* $(M+H)^+$; Found, 345.1705 *m/z*.

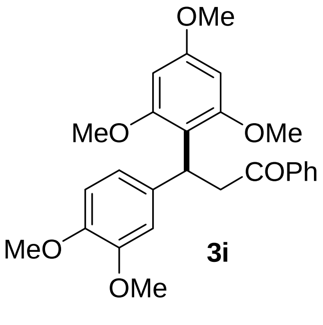

3i

3-(3,4-Dimethoxy-phenyl)-1-phenyl-3-(2,4,6-trimethoxy-phenyl)-propan-1-one. Yield = 86%, Column purified with EtOAc: Pet Ether = 1:4, White amorphous solid, mp 145-146 °C.

^{1}H NMR ($CDCl_3$, 400 MHz): δ 7.93 (d, *J* = 8.0 Hz, 2H), 7.55-7.48 (m, 1H), 7.47-7.42 (m, 2H), 6.92 (s, 1H), 6.85 (d, *J* = 8.0 Hz, 1H), 6.71 (d, *J* = 8.0 Hz, 1H), 6.10 (s, 2H), 5.30 (t, *J* = 8.0 Hz, 1H), 3.85 (dd, *J* = 8.8, 2.8 Hz, 2H), 3.80 (s, 3H), 3.79 (s, 3H), 3.77 (s, 3H), 3.73 (s, 6H).

^{13}C-NMR ($CDCl_3$, 100 MHz): δ 199.9, 159.6, 158.9 (2C), 148.3, 146.9, 137.5, 132.6, 128.0 (2C), 127.9, 127.8 (2C), 119.5, 113.1, 111.6, 110.7, 91.3 (2C), 55.81 (2C), 55.74, 55.72, 55.2, 42.68, 34.9.

HRMS (EI) calcd for $C_{26}H_{28}O_6$, 437.1964 *m/z* $(M+H)^+$; Found, 437.1969 *m/z.*

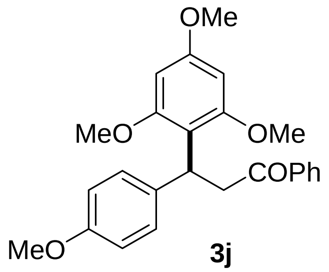

3j

3-(4-Methoxy-phenyl)-1-phenyl-3-(2,4,6-trimethoxy-phenyl)-propan-1-one. Yield = 75%, Column purified with EtOAc: Pet Ether = 1:5, White crystalline needles (Recrystallised from EA-Petroleum ether mixture), mp 141-142 °C.

^{1}H NMR ($CDCl_3$, 200 MHz): δ 7.98 (d, *J* = 8.0 Hz, 2H), 7.57-7.53 (m, 1H), 7.47-7.43 (m, 2H), 7.30 (d, *J* = 8.0 Hz, 2H), 6.81 (d, *J* = 8.0 Hz, 2H), 6.13 (s, 2H), 5.37 (t, *J* = 7.6 Hz, 1H), 3.91 (dd, *J* = 7.4, 1.9 Hz, 2H), 3.79 (s, 3H), 3.77 (s, 3H), 3.75 (s, 6H).

^{13}C-NMR ($CDCl_3$, 100 MHz): δ 199.7, 159.5, 158.7 (2C), 157.2, 137.3, 136.7, 132.4, 128.4 (2C), 128.2 (2C), 127.9 (2C), 113.1 (3C), 91.1 (2C), 55.6 (3C), 55.0, 42.4, 34.3.

HRMS (EI) calcd for $C_{25}H_{26}O_5$, 407.1859 *m/z* $(M+H)^+$; Found, 407.1865 *m/z.*

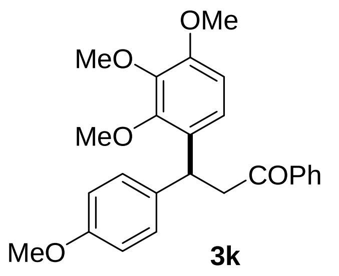

3-(4-Methoxy-phenyl)-1-phenyl-3-(2,3,4-trimethoxy-phenyl)-propan-1-one. Yield = 74%, Column purified with EtOAc: Pet Ether = 1:5, White amorphous solid, mp 138-140 °C.

^{1}H NMR ($CDCl_3$, 200 MHz): δ 7.96 (d, *J* = 7.6 Hz, 2H), 7.55-7.41 (m, 3H), 7.18 (d, *J* = 8.0 Hz, 2H), 6.78 (m, 3H), 6.60 (d, *J* = 8.0 Hz, 1H), 5.00 (t, *J* = 8.0 Hz, 1H), 3.84 (s, 3H), 3.82 (s, 3H), 3.78 (s, 3H), 3.68 (s, 3H), 3.66 (m, 2H).

^{13}C-NMR ($CDCl_3$, 100 MHz): δ 198.6, 158.0, 152.3, 151.6, 142.5, 136.44, 136.0, 133.0, 131.2, 130.3, 128.9, 128.6, 128.2, 124.2, 122.0, 113.8, 113.6, 107.0, 106.5, 60.8, 60.5, 56.0, 55.3, 44.4, 39.4.

HRMS (EI) calcd for $C_{25}H_{26}O_5$, 407.1859 *m/z* $(M+H)^+$; Found, 407.1863 *m/z*.

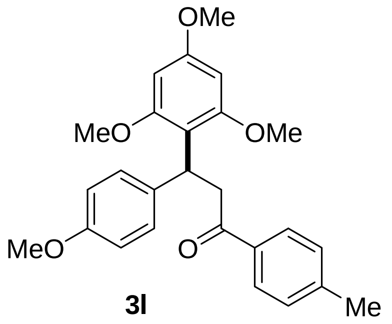

3-(4-Methoxy-phenyl)-1-p-tolyl-3-(2,4,6-trimethoxy-phenyl)-propan-1-one. Yield = 86%, Column purified with EtOAc: Pet Ether = 1:5, White solid, mp 151-153 °C.

^{1}H NMR ($CDCl_3$, 200 MHz): δ 7.88 (d, *J* = 8.0 Hz, 2H), 7.27 (d, *J* = 8.6 Hz, 2H), 7.25 (d, *J* = 8.0 Hz, 2H), 6.78 (d, *J* = 8.0 Hz, 2H), 6.12 (s, 2H), 5.34 (t, *J* = 8.0 Hz, 1H), 3.86 (d, *J* = 7.6 Hz, 2H), 3.79 (s, 3H), 3.77 (s, 3H), 3.75 (s, 6H), 2.42 (s, 3H).

^{13}C-NMR ($CDCl_3$, 100 MHz): δ 199.5, 159.7, 159.0, 157.4, 143.3, 137.1, 135.1, 129.1 (2C), 128.7 (2C), 128.3 (2C), 128.6, 113.6, 113.3 (2C), 91.37 (2C), 55.85 (3C), 55.31, 42.5, 34.5, 21.7.

HRMS (EI) calcd for $C_{26}H_{28}O_5$, 421.2015 *m/z* $(M+H)^+$; Found, 421.2019 *m/z.*

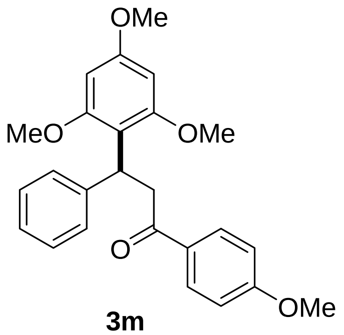

3m

1-(4-Methoxy-phenyl)-3-phenyl-3-(2,4,6-trimethoxy-phenyl)-propan-1-one. Yield = 71%, Column purified with EtOAc: Pet Ether = 1:4, Colorless crystalline needles solid (Recrystallised from EA-Petroleum ether mixture), 134-135 °C.

^{1}H NMR ($CDCl_3$, 200 MHz): δ 7.98 (d, *J* = 8.4 Hz, 2H), 7.37-7.34 (m, 2H), 7.25-7.22 (m, 2H), 7.16-7.11 (m, 1H), 6.92 (d, *J* = 8.4 Hz, 2H), 6.12 (s, 2H), 5.41 (t, *J* = 8.0 Hz, 1H), 3.88 (d, *J* = 8.0 Hz, 2H), 3.86 (s, 3H), 3.79 (s, 3H), 3.73 (s, 6H).

^{13}C-NMR ($CDCl_3$, 100 MHz): δ 198.3, 163.1, 159.7, 159.0 (2C), 144.9, 130.6, 130.4 (2C), 127.8 (2C), 127.7 (2C), 125.4, 113.5 (2C), 113.3, 91.3 (2C), 55.78 (2C), 55.44, 55.23, 41.9, 35.2.

HRMS (EI) calcd for $C_{25}H_{26}O_5$, 407.1859 *m/z* $(M+H)^+$; Found, 407.1864 *m/z.*

<u>Spectral Data of all the Friedel-Crafts Alkylated Products 5a-5d</u>

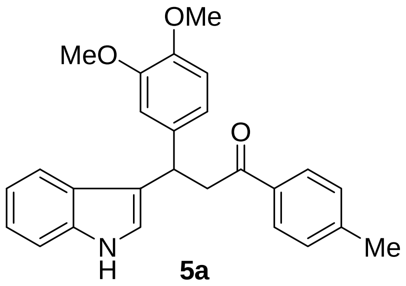

5a

3-(3,4-Dimethoxy-phenyl)-3-(1*H*-indol-3-yl)-1-*p*-tolyl-propan-1-one. Yellow amorphous solid (Recrystallised from MeOH), Yield = 87%, Column purified with EtOAc: Pet Ether = 1:6, mp 152 °C.

^{1}H NMR ($CDCl_3$, 400 MHz): δ 8.10 (bs, 1H), 7.87 (d, *J* = 8.0 Hz, 2H), 7.48 (d, *J* = 8.0 Hz, 1H), 7.34 (d, *J* = 8.0 Hz, 1H), 7.27 (d, *J* = 8.0 Hz, 2H), 7.19-7.15 (m, 1H), 7.05 (t, *J* = 7.6 Hz. 1H), 6.97 (s, 1H), 6.96-6.88 (m, 2H), 6.76 (d, *J* = 8.0 Hz, 1H), 5.04 (t, *J* = 6.8 Hz, 1H), 4.06 (d, *J* = 16.0 Hz, 1H), 3.90 (d, *J* = 16.0 Hz, 1H), 3.87 (s, 3H), 3.83 (s, 3H), 2.38 (s, 3H).

^{13}C-NMR ($CDCl_3$, 100 MHz): δ 198.5, 148.7, 147.3, 143.8, 136.9, 136.7, 134.7, 129.3 (2C), 128.2 (2C), 126.6, 122.1, 121.4, 119.57, 119.54, 119.5, 119.3, 111.5, 111.2, 111.1, 55.8 (2C), 45.2, 38.0, 22.0.

HRMS (EI) calcd for $C_{26}H_{25}NO_3$, 400.1913 *m/z* $(M+H)^+$; Found, 400.1911 *m/z.*

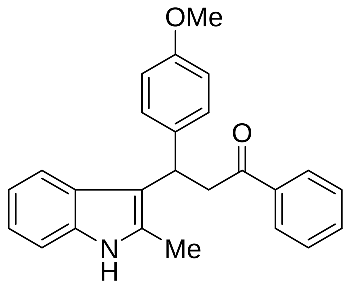

5b

3-(4-Methoxy-phenyl)-3-(2-methyl-1*H*-indol-3-yl)-1-phenyl-propan-1-one. Yellow amorphous solid (Recrystallised from MeOH), Yield = 93%, Column purified with EtOAc: Pet Ether = 1:5, mp 143 °C.

^{1}H NMR ($CDCl_3$, 400 MHz): δ 7.91 (d, *J* = 8.0 Hz, 2H), 7.53-7.48 (m, 2H), 7.39 (t, *J* = 7.8 Hz, 2H), 7.33-7.28 (m, 2H), 7.24-7.17 (m, 1H), 7.11-6.99 (m, 2H), 6.82 (d, *J* = 8.0 Hz, 2H), 5.07 (t, *J* = 7.2 Hz, 1H), 3.93 (dd, *J* = 7.2, 4.0 Hz, 2H), 3.76 (s, 3H), 2.37 (s, 3H).

^{13}C-NMR ($CDCl_3$, 100 MHz): δ 199.4, 157.7, 137.2, 136.4, 135.6, 133.0, 131.7, 128.5 (3C), 128.1 (3C), 127.5, 120.7, 119.1, 113.8 (2C), 113.7 (2C), 110.5, 55.26, 43.9, 36.1, 12.1.

HRMS (EI) calcd for $C_{25}H_{23}NO_2$, 370.1807 *m/z* $(M+H)^+$; Found, 370.1816 *m/z.*

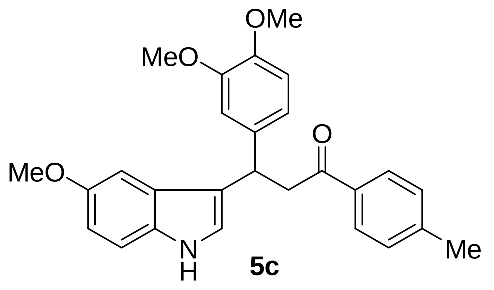

3-(3,4-Dimethoxy-phenyl)-3-(5-methoxy-1*H*-indol-3-yl)-1-*p*-tolyl-propan-1-one. Yield = 91%, Column purified with EtOAc: Pet Ether = 1:4, Brown needles solid (Recrystallised from MeOH), mp 154 °C.

^{1}H NMR ($CDCl_3$, 400 MHz): δ 8.04 (s, 1H), 7.94-7.78 (m, 2H), 7.34-7.11 (m, 3H), 7.00-6.71 (m, 6H), 4.97 (t, *J* = 7.2 Hz, 1H), 3.82 (s, 3H), 3.77 (s, 3H), 3.75 (s, 3H), 3.74-3.67 (m, 2H), 2.39 (s, 3H).

^{13}C-NMR ($CDCl_3$, 100 MHz): δ 198.7, 153.8, 148.9, 147.5, 143.9, 137.0, 134.8, 131.9, 129.4 (2C), 128.3 (2C), 127.1, 122.3, 119.6, 119.3, 112.2, 111.9, 111.7, 111.2, 101.7, 56.0, 55.9, 55.9, 48.2, 38.1, 21.7.

HRMS (EI) calcd for $C_{27}H_{27}NO_4$, 430.2018 *m/z* $(M+H)^+$; Found, 430.2021 *m/z*.

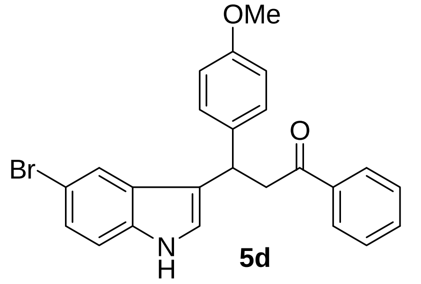

3-(5-Bromo-1H-indol-3-yl)-3-(4-methoxy-phenyl)-1-phenyl-propan-1-one.[1] Yield = 86%, Column purified with EtOAc: Pet Ether = 1:4, Brown crystalline solid (Recrystallised from MeOH), mp 153 °C.

^{1}H NMR ($CDCl_3$, 400 MHz): δ 8.13 (bs, 1H), 7.92 (d, *J* = 8.2 Hz, 2H), 7.58-7.50 (m, 2H), 7.50-7.39 (m, 2H), 7.24-7.12 (m, 4H), 6.93 (s, 1H), 6.82 (d, *J* = 8.6 Hz, 2H), 4.95 (t, *J* = 7.2 Hz, 1H), 3.74 (s, 3H), 3.74-3.67 (m, 2H).

^{13}C-NMR ($CDCl_3$, 100 MHz): δ 198.8, 158.2, 137.1, 136.0, 135.4, 133.2, 129.5, 128.7 (2C), 128.5, 128.2 (2C), 125.1, 124.9, 122.6, 122.3, 122.1, 119.4, 114.1, 113.9, 112.8, 55.4, 45.5, 37.4.

HRMS (EI) calcd for $C_{24}H_{20}BrNO_2$, 434.0756 *m/z* $(M+H)^+$; Found, 434.0757 *m/z.*

Reference 1: *Indian Journal of Chemistry*, Volume 54B, **2015**, 240-244

<u>Copy of ^{1}H NMR and ^{13}C Spectra of Friedel-Crafts Alkylated Products 3a-3m</u>

^{1}H-NMR of compound **3a**

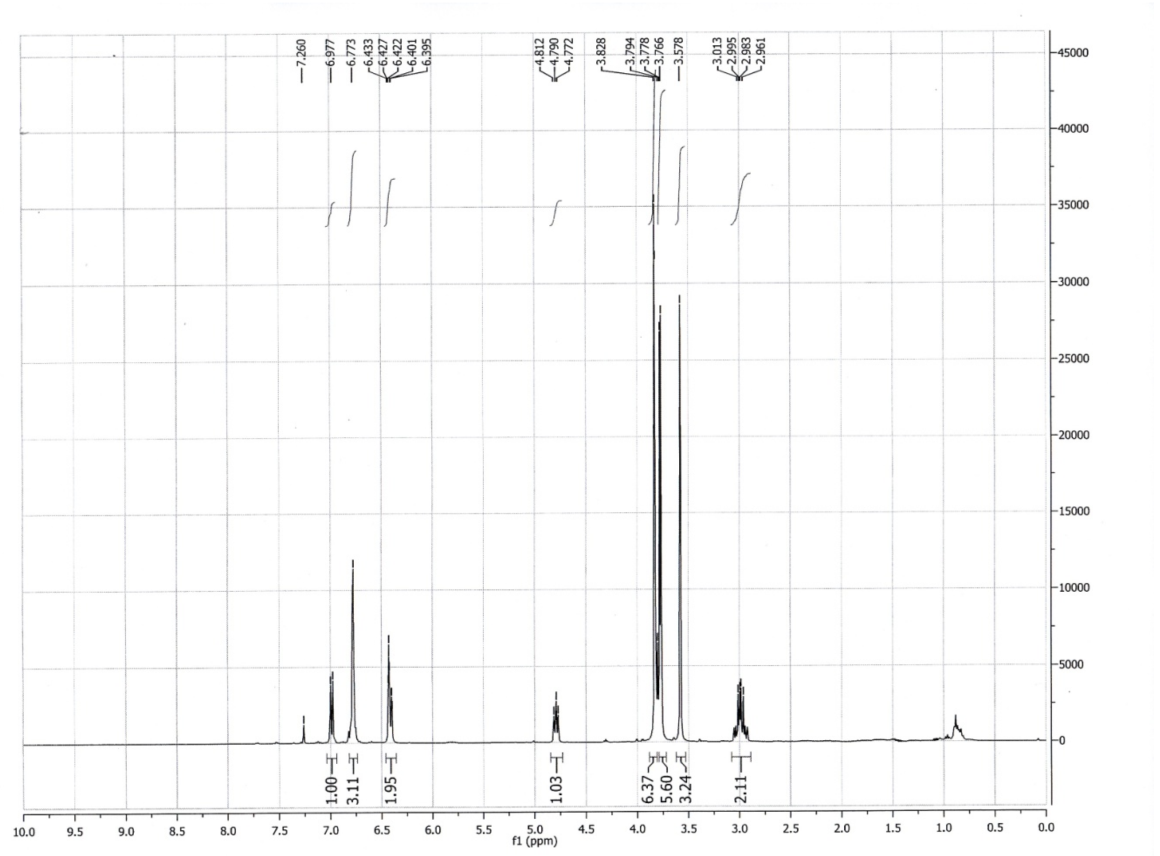

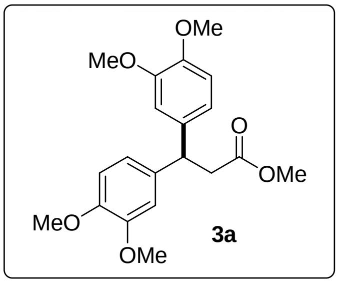

^{13}C-NMR of compound **3a**

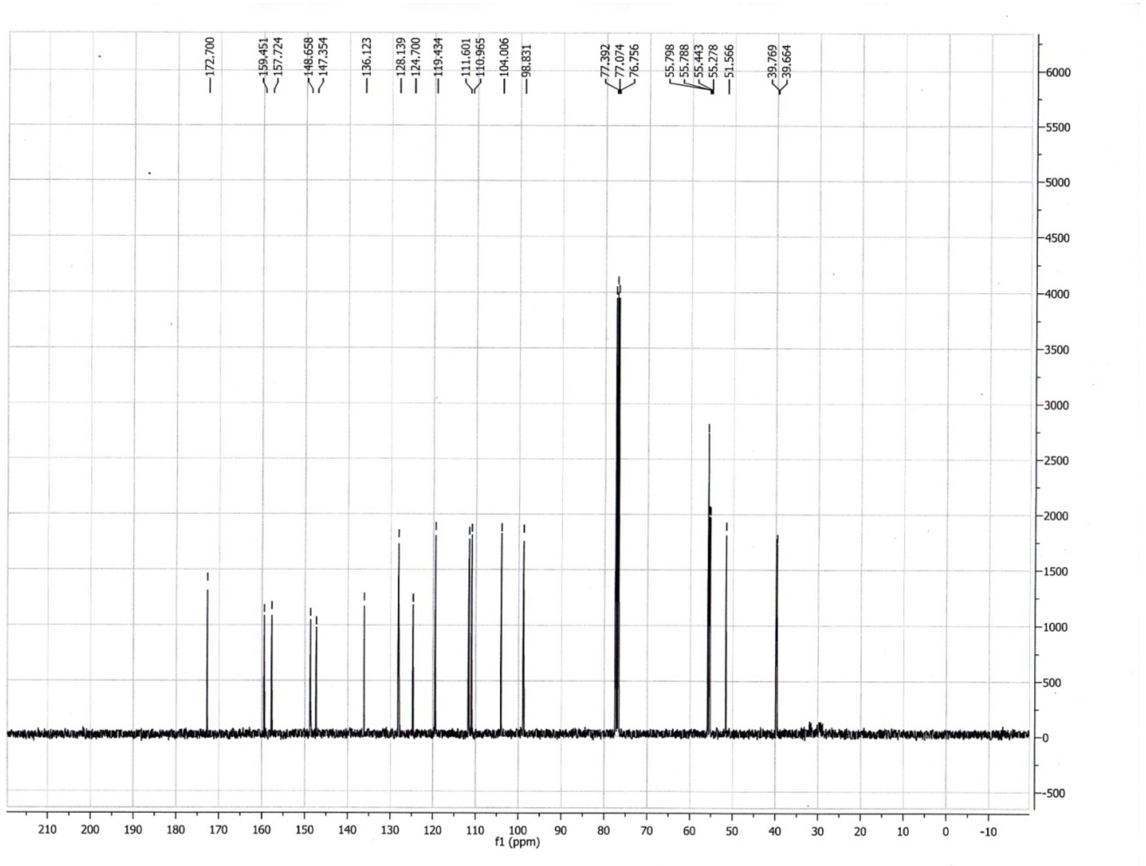

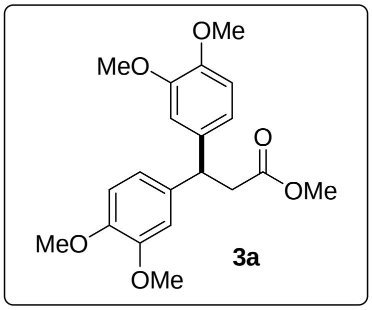

^{1}H-NMR of compound **3b**

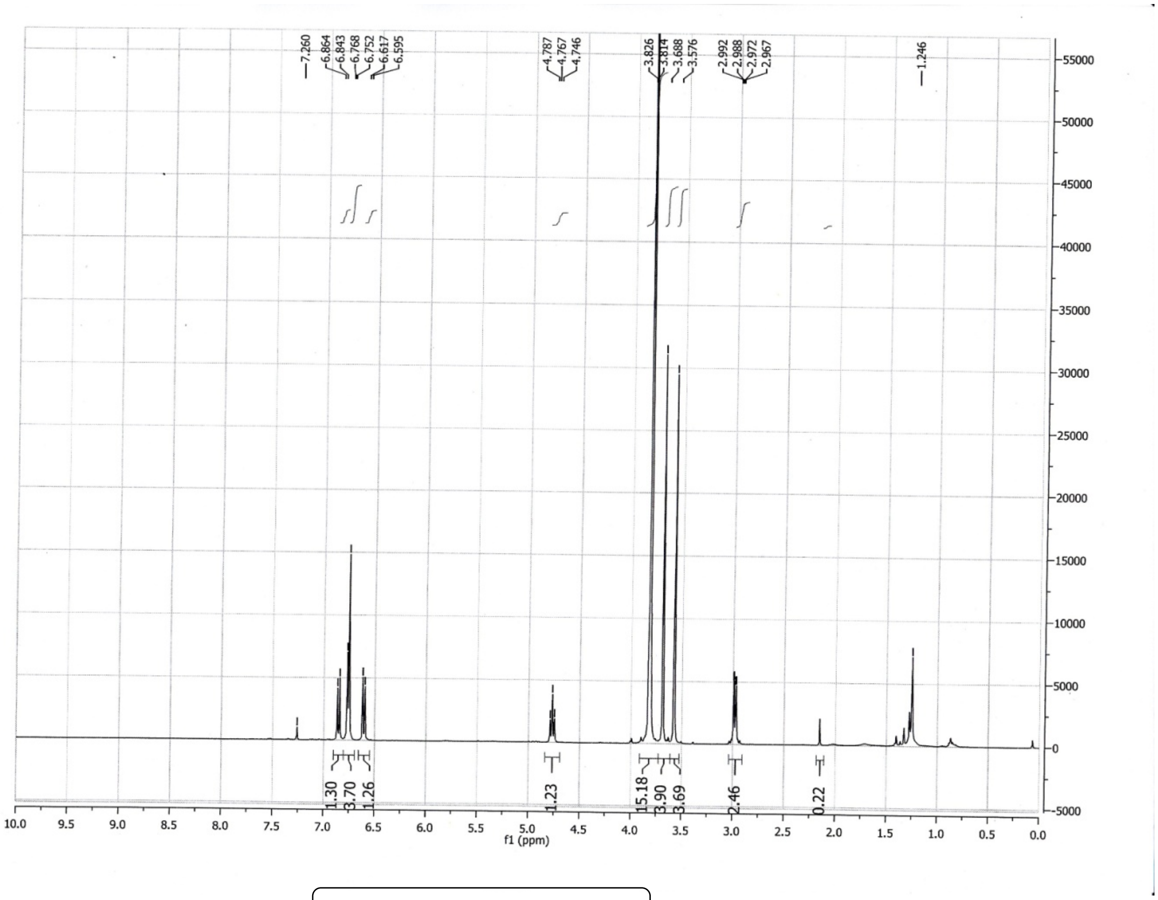

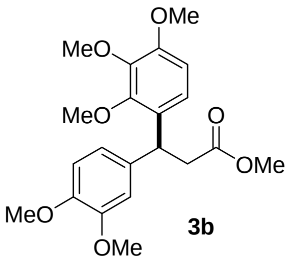

3b

^{13}C-NMR of compound **3b**

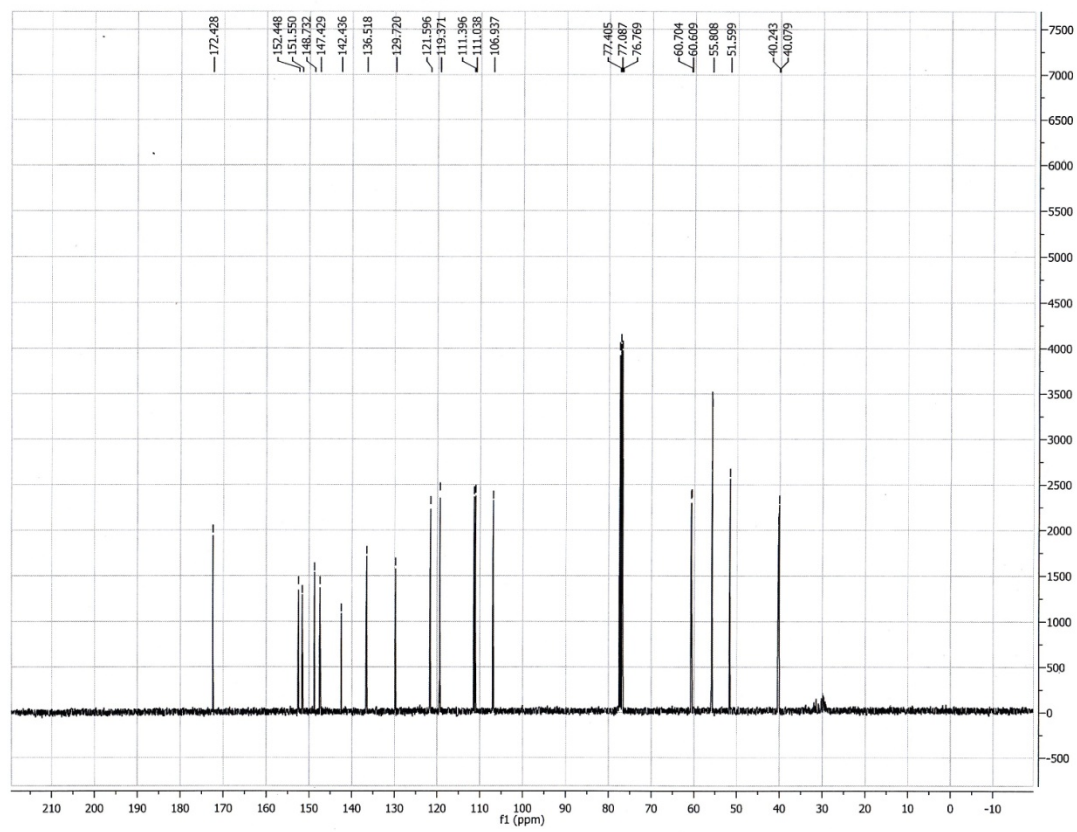

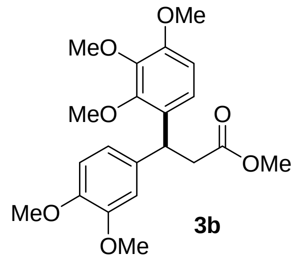

3b

^{1}H-NMR of compound **3c**

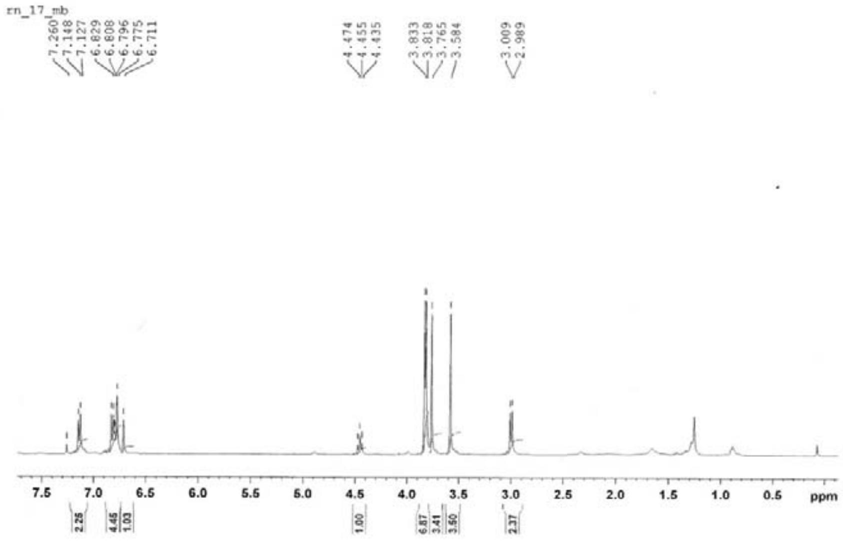

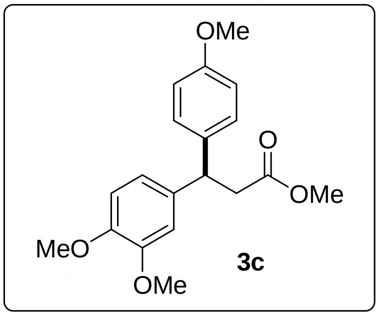

^{13}C-NMR of compound **3c**

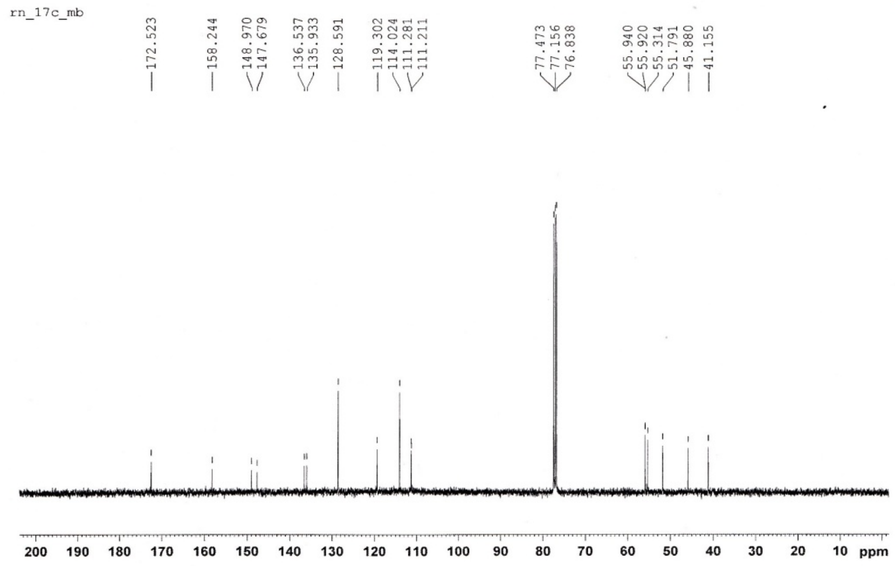

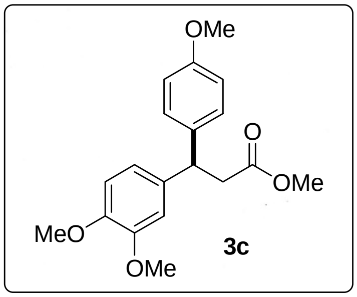

3c

DEPT-135 of compound **3c**

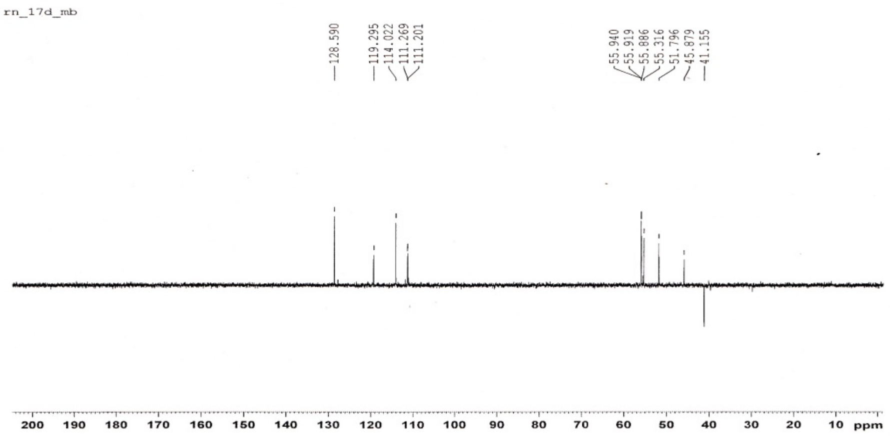

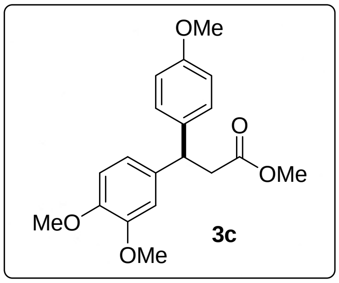

^{1}H-NMR of compound **3d**

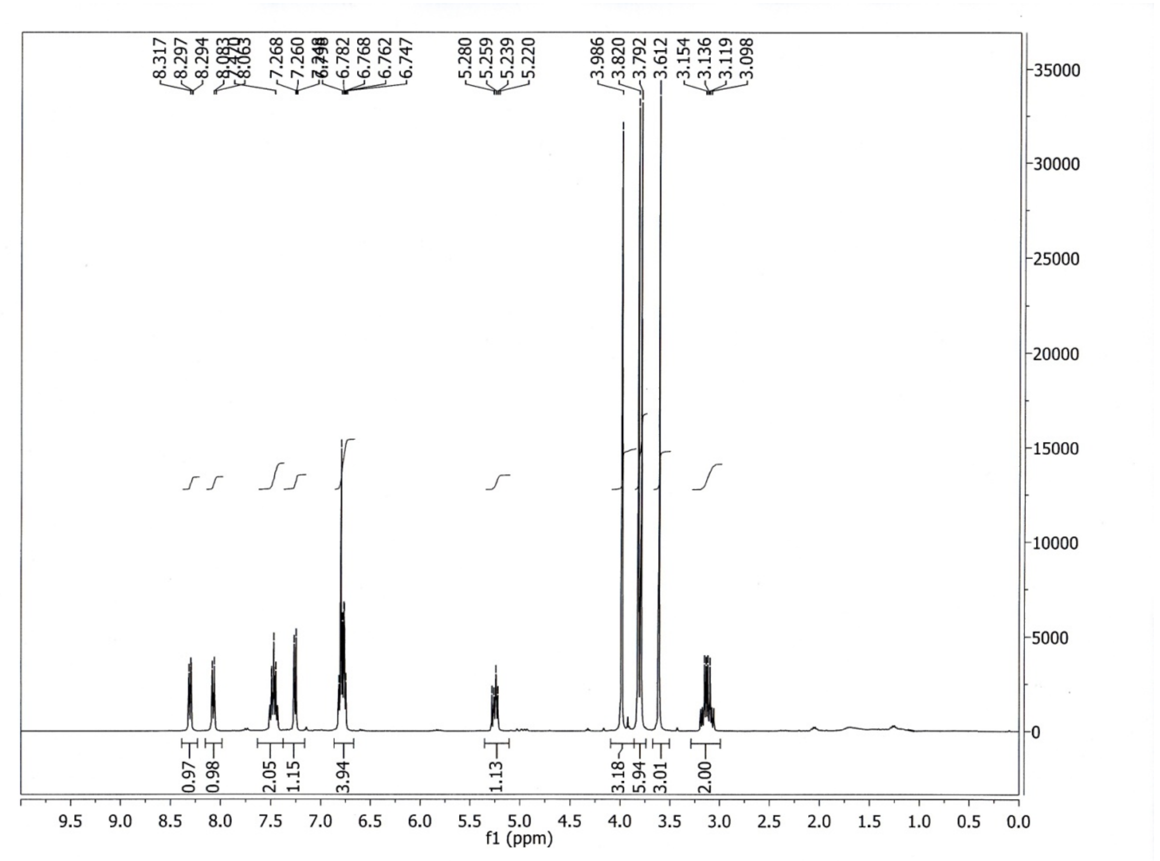

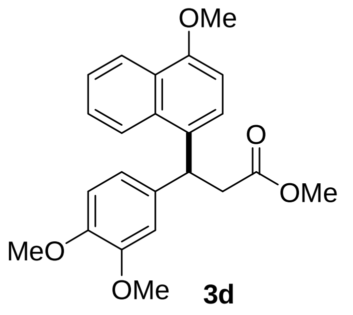

3d

^{13}C-NMR of compound **3d**

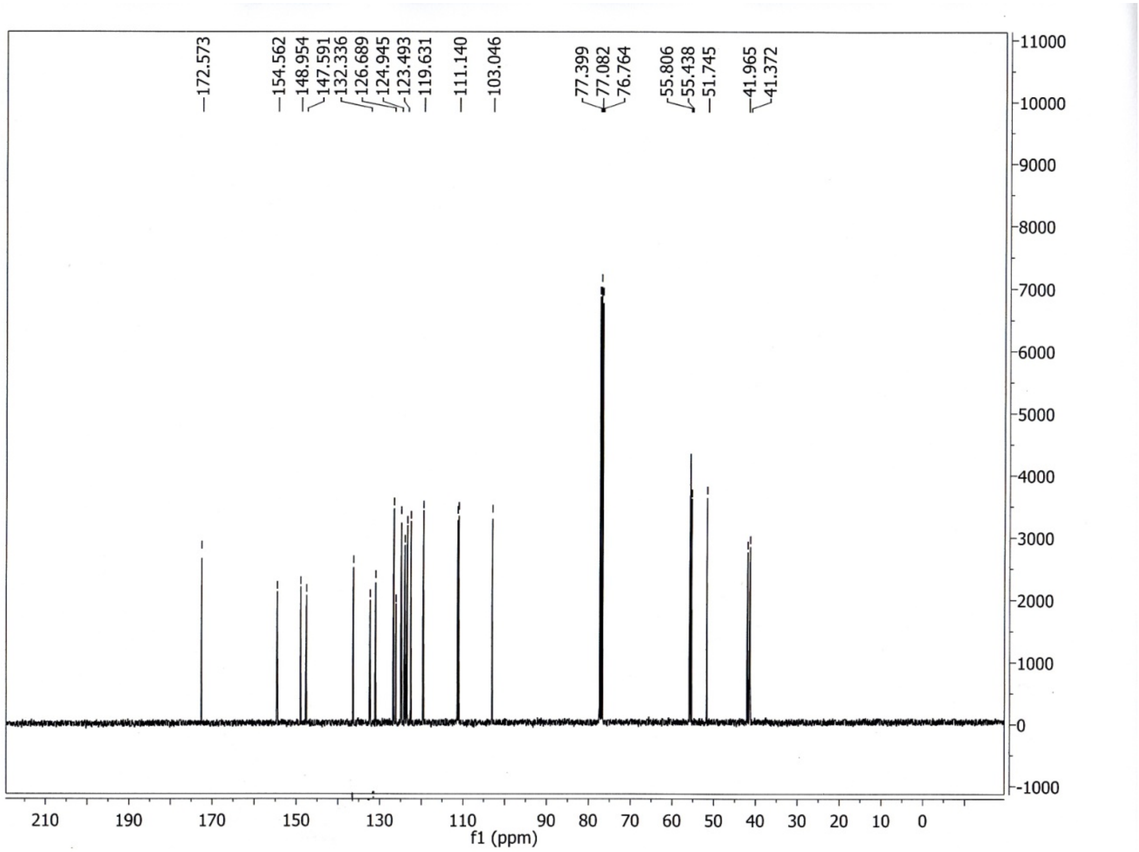

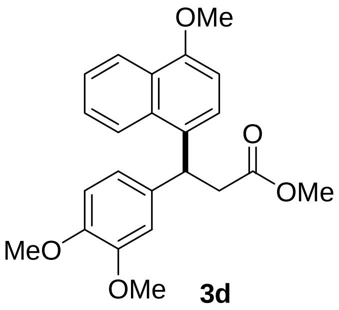

3d

^{1}H-NMR of compound **3e**

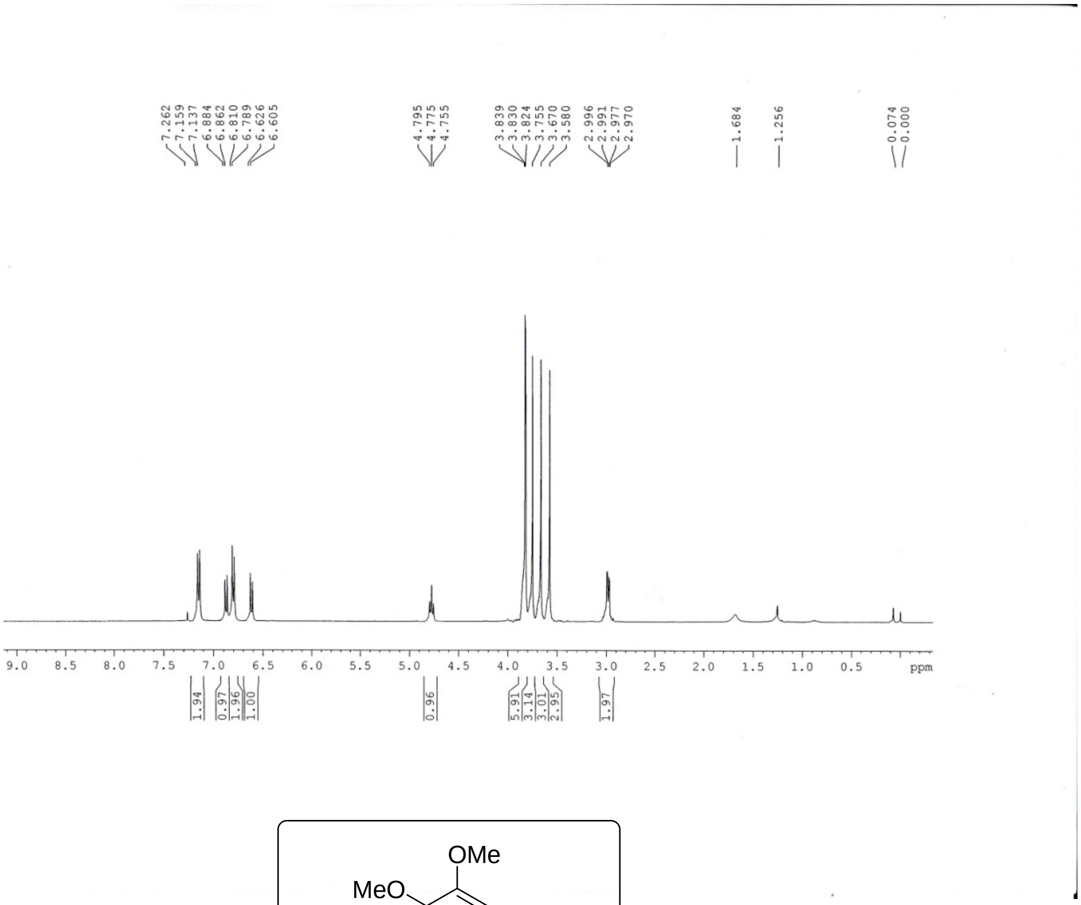

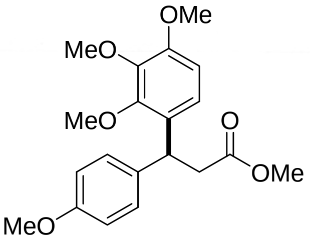

3e

^{13}C-NMR of compound **3e**

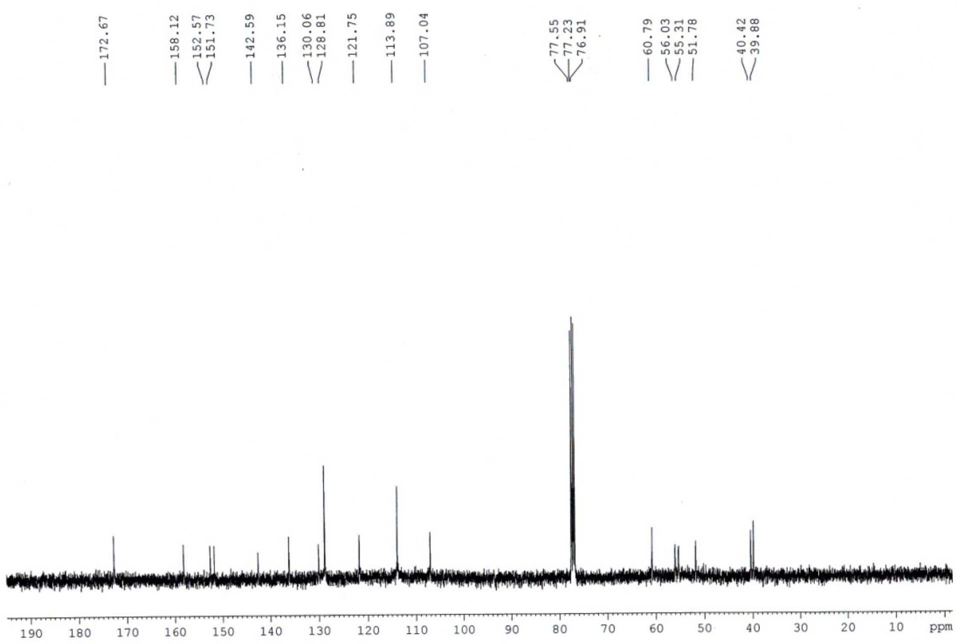

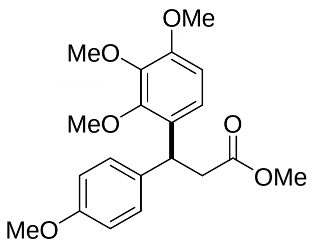

3e

DEPT-135 of compound **3e**

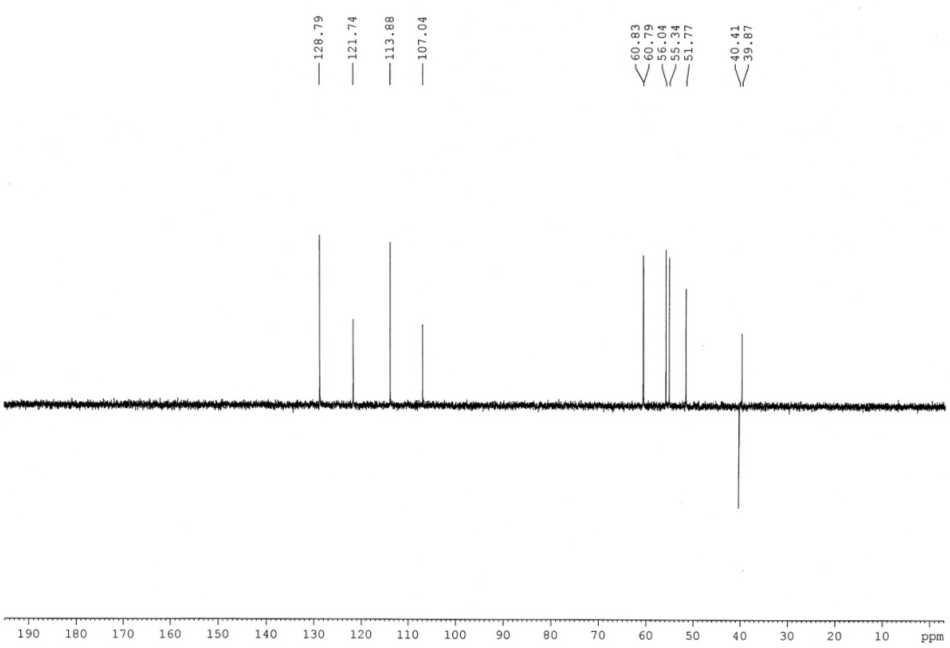

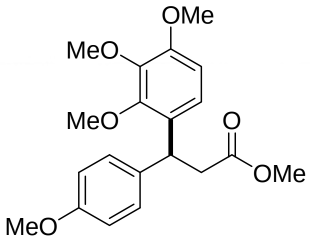

3e

^{1}H-NMR of compound **3f**

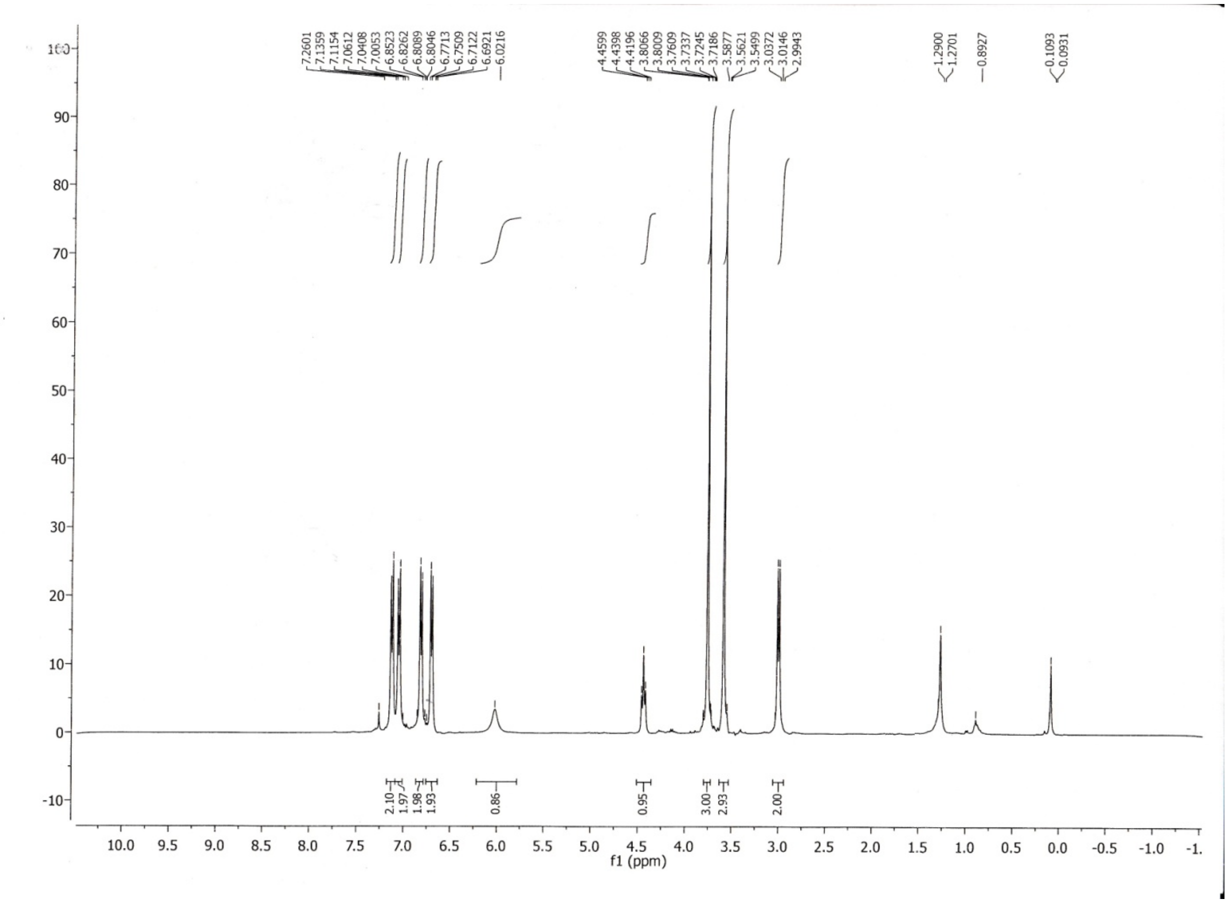

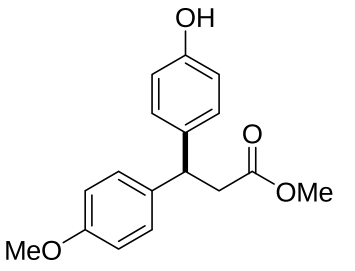

3f

^{13}C-NMR of compound **3f**

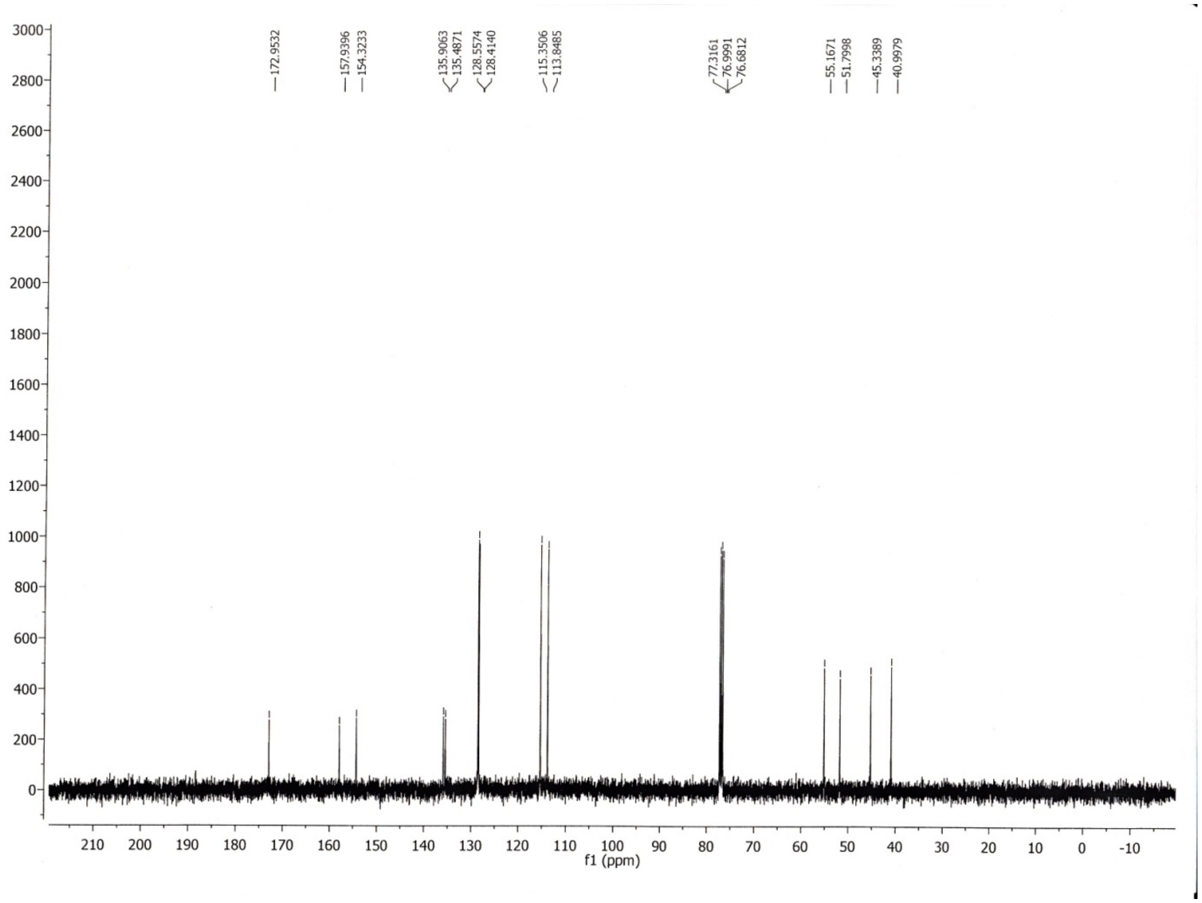

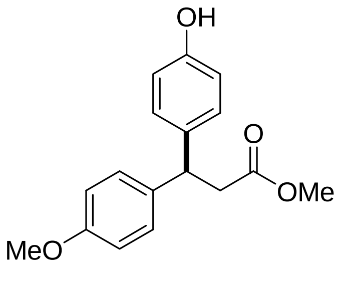

3f

^{1}H-NMR of compound **3g**

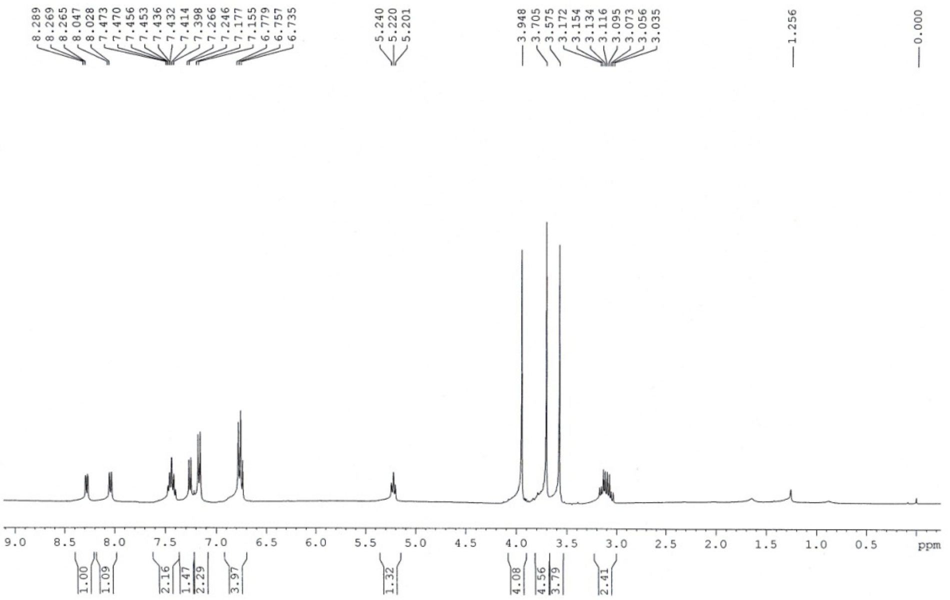

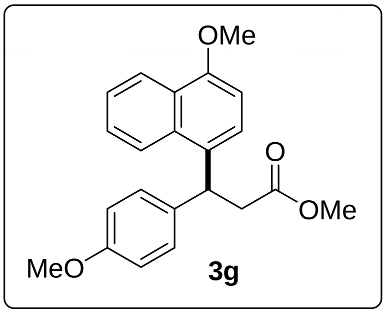

^{13}C-NMR of compound **3g**

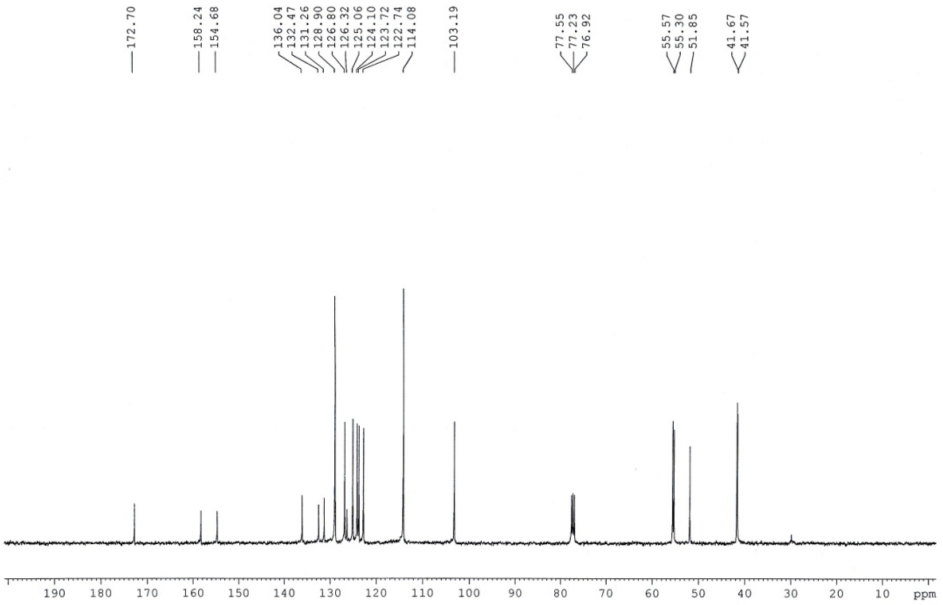

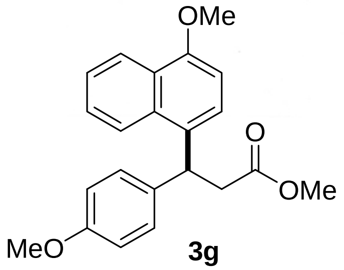

3g

DEPT-135 of compound **3g**

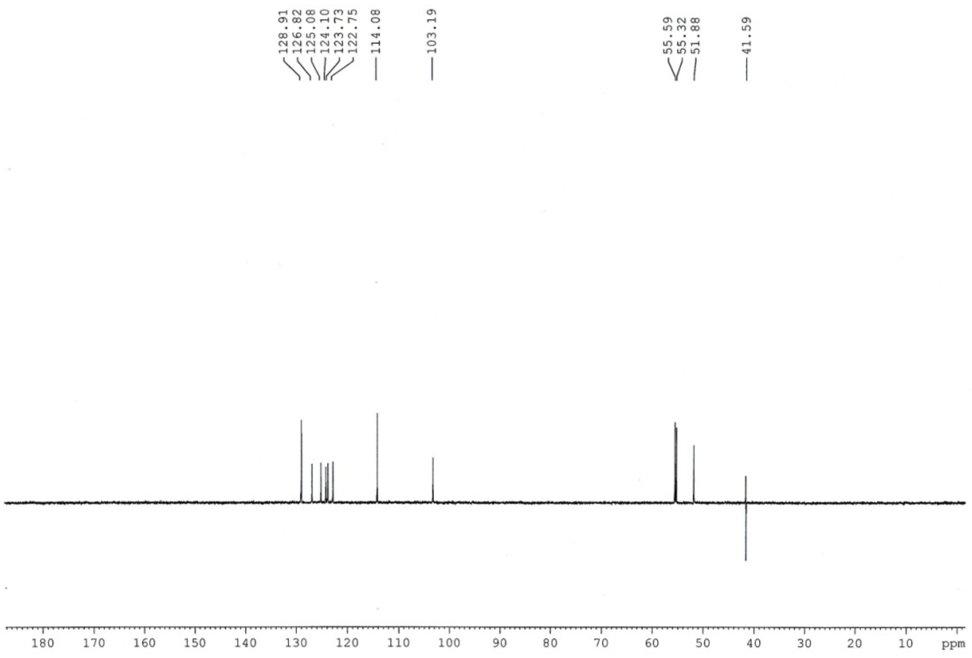

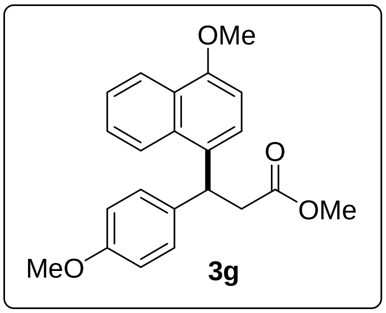

^{1}H-NMR of compound **3h**

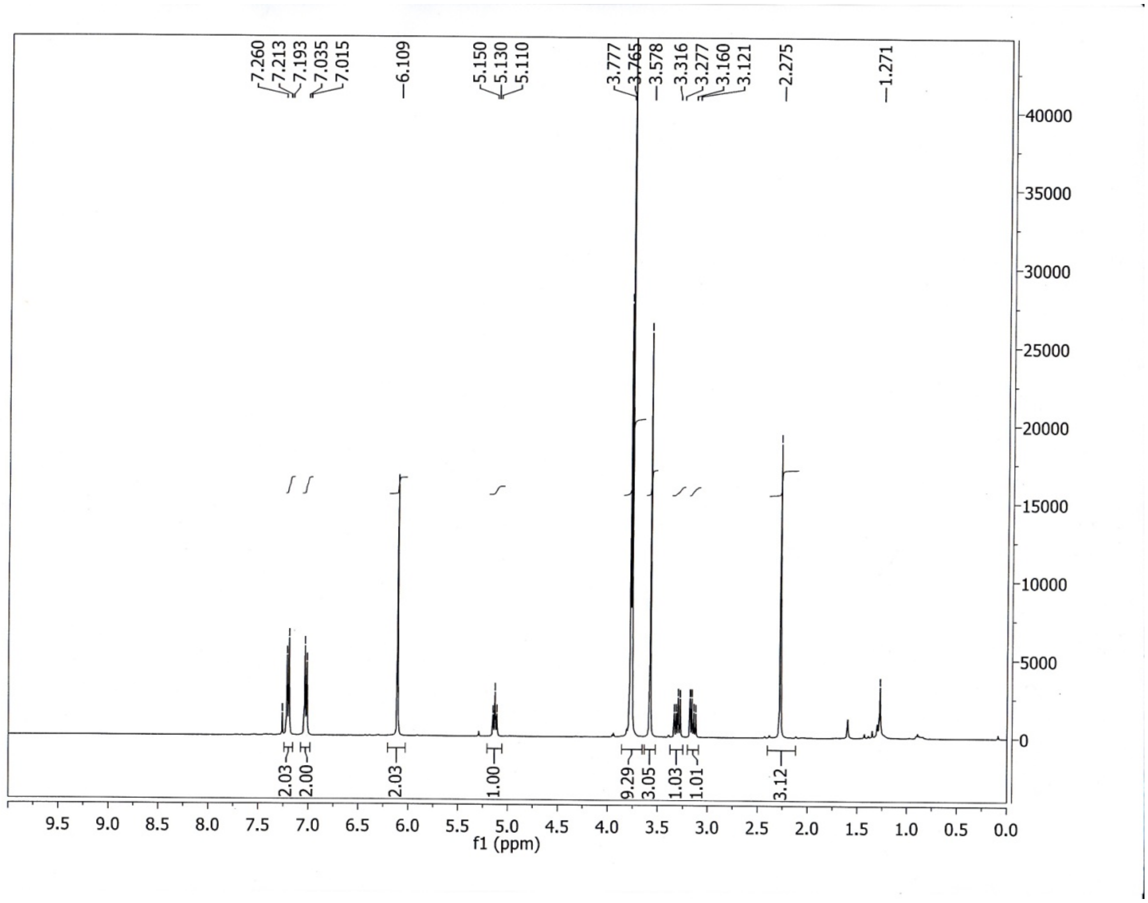

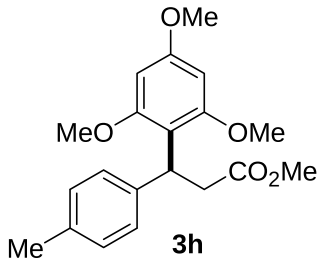

3h

^{13}C-NMR of compound **3h**

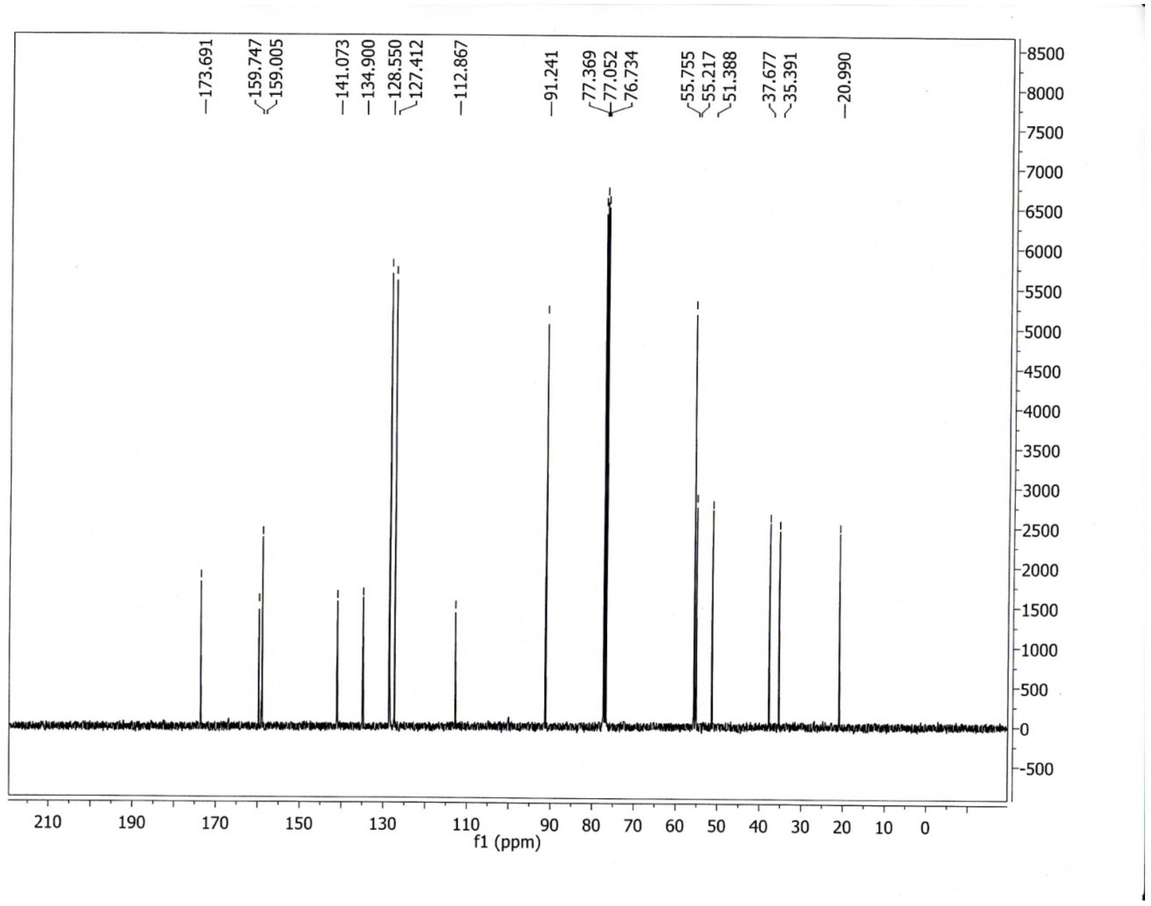

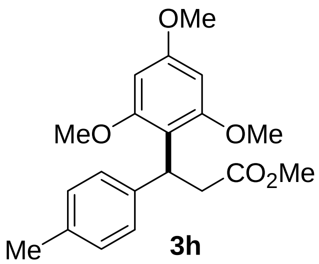

3h

^{1}H-NMR of compound **3i**

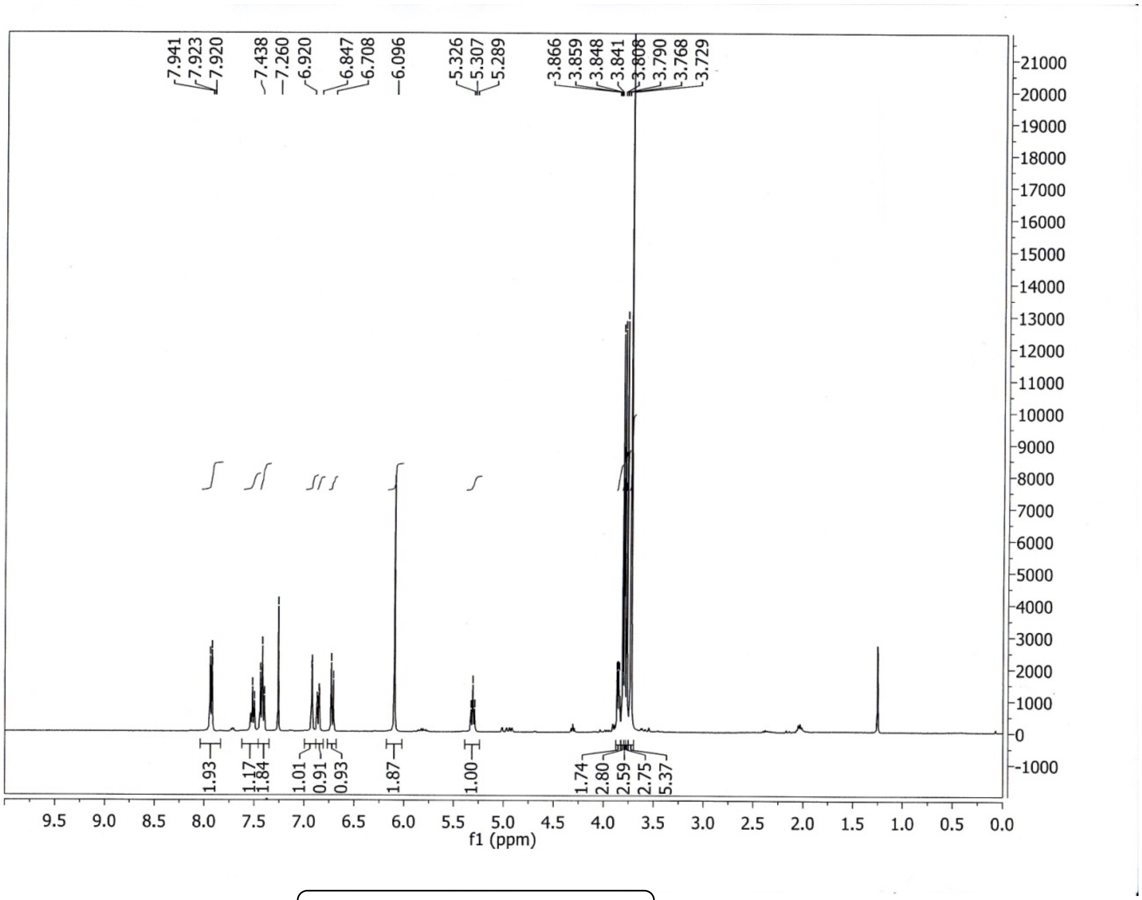

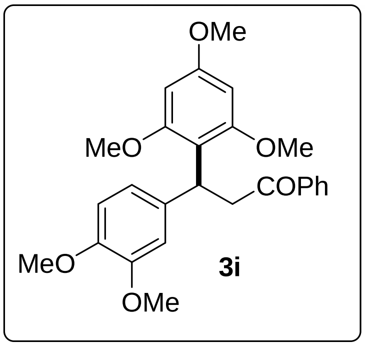

^{13}C-NMR of compound **3i**

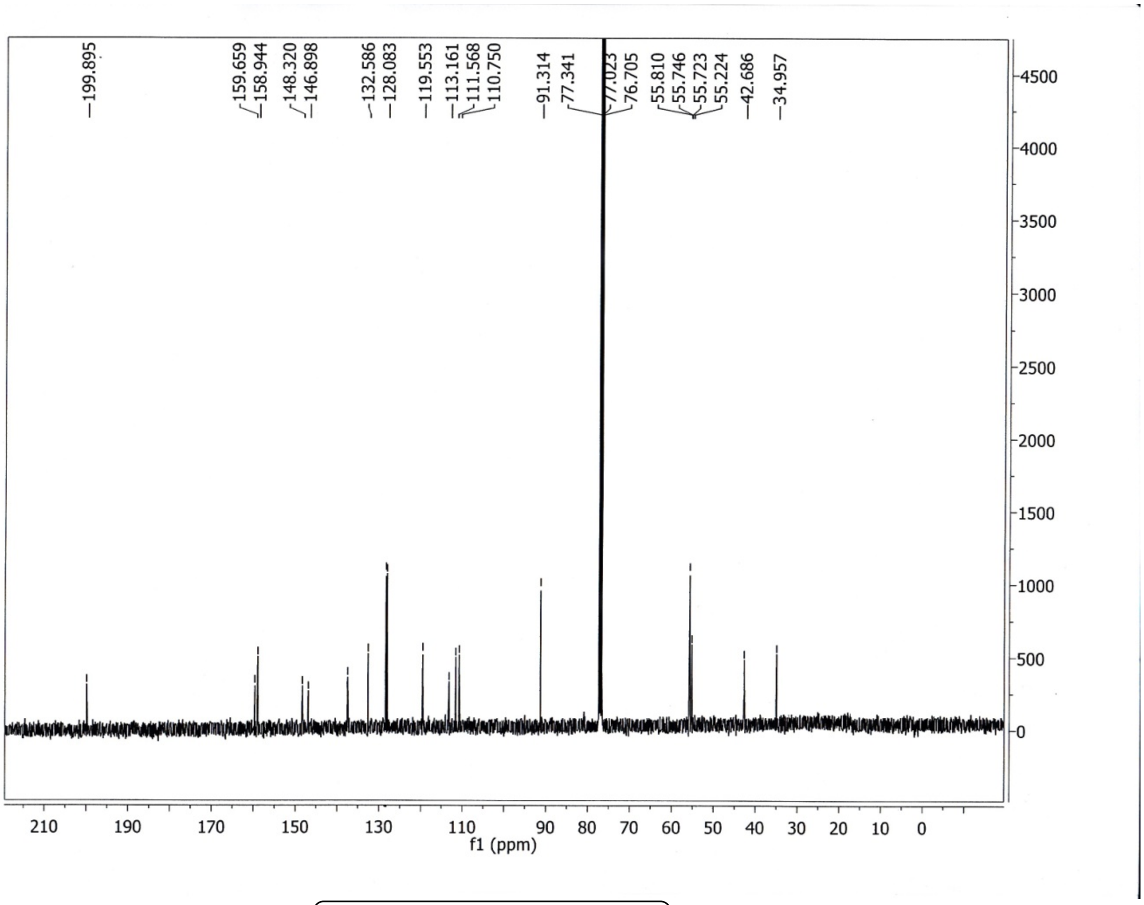

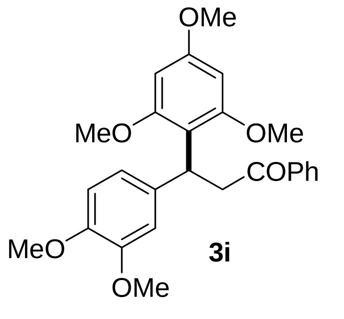

3i

^{1}H-NMR of compound **3j**

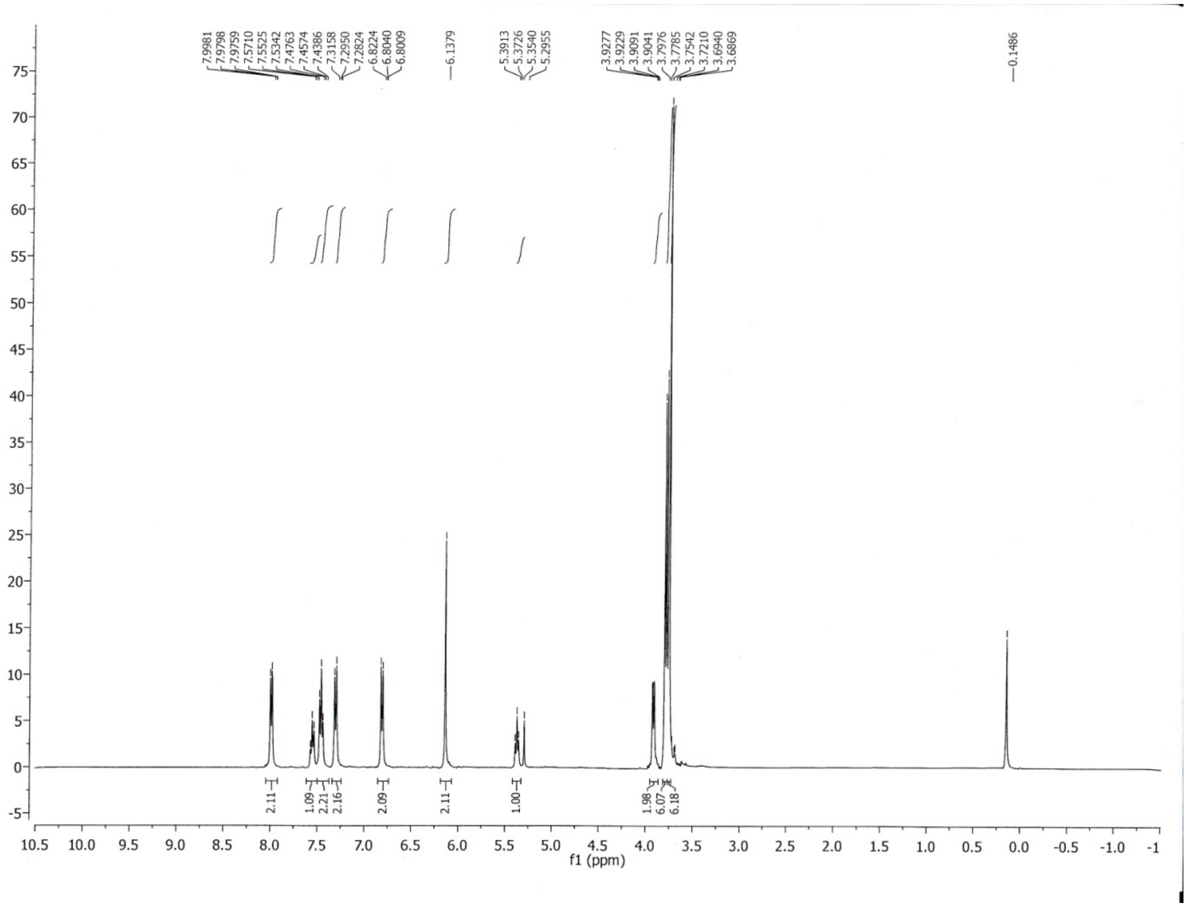

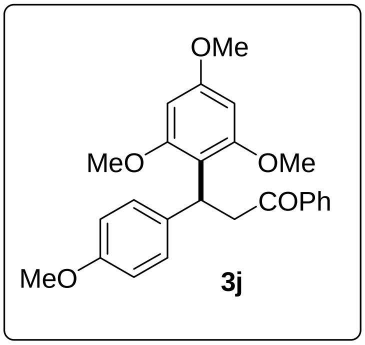

^{13}C-NMR of compound **3j**

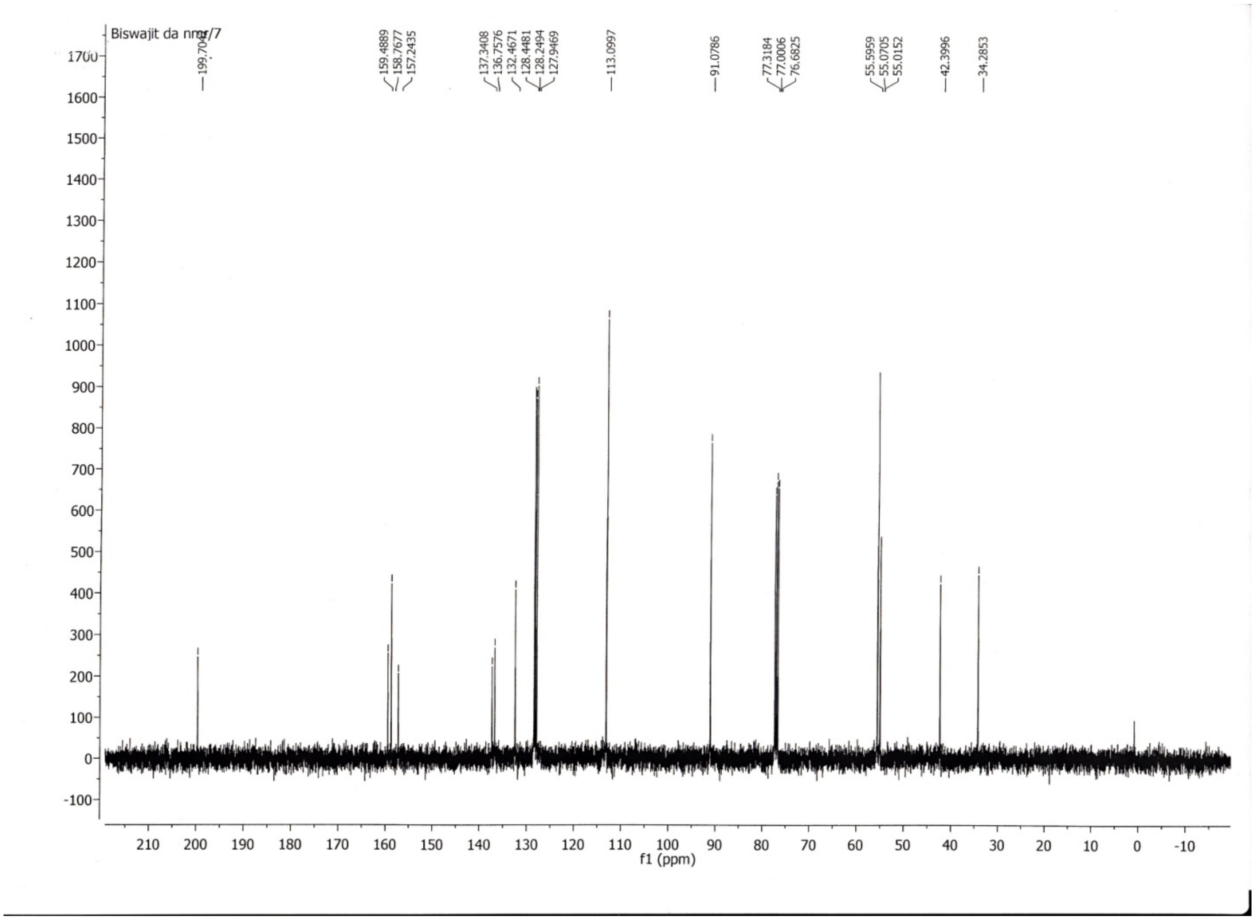

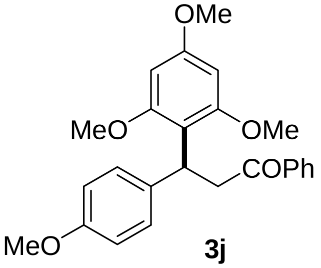

3j

^{1}H-NMR of compound **3k**

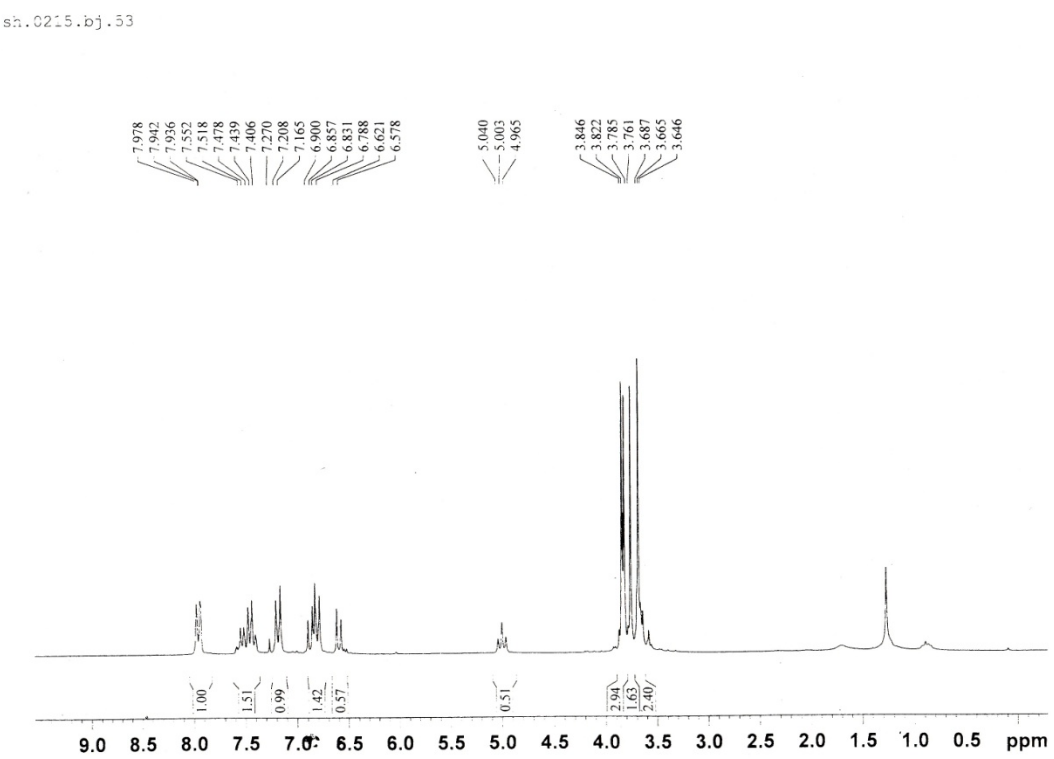

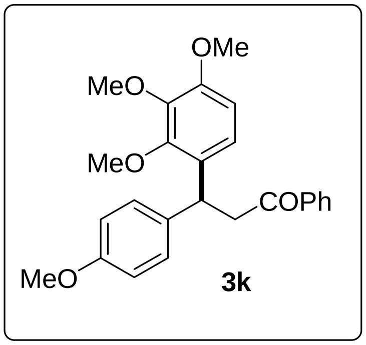

^{13}C-NMR of compound **3k**

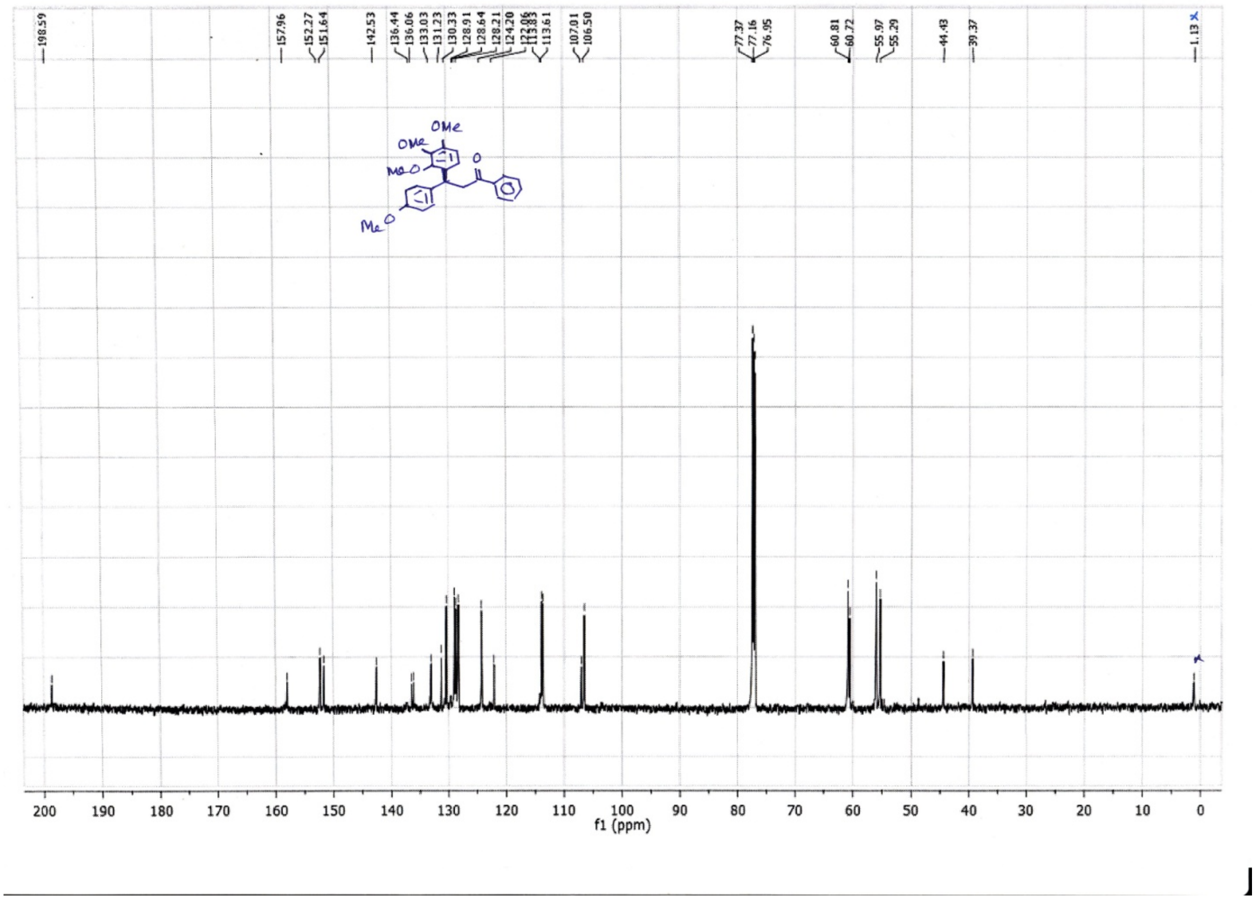

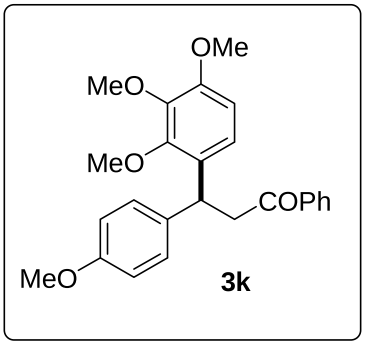

^{1}H-NMR of compound **3l**

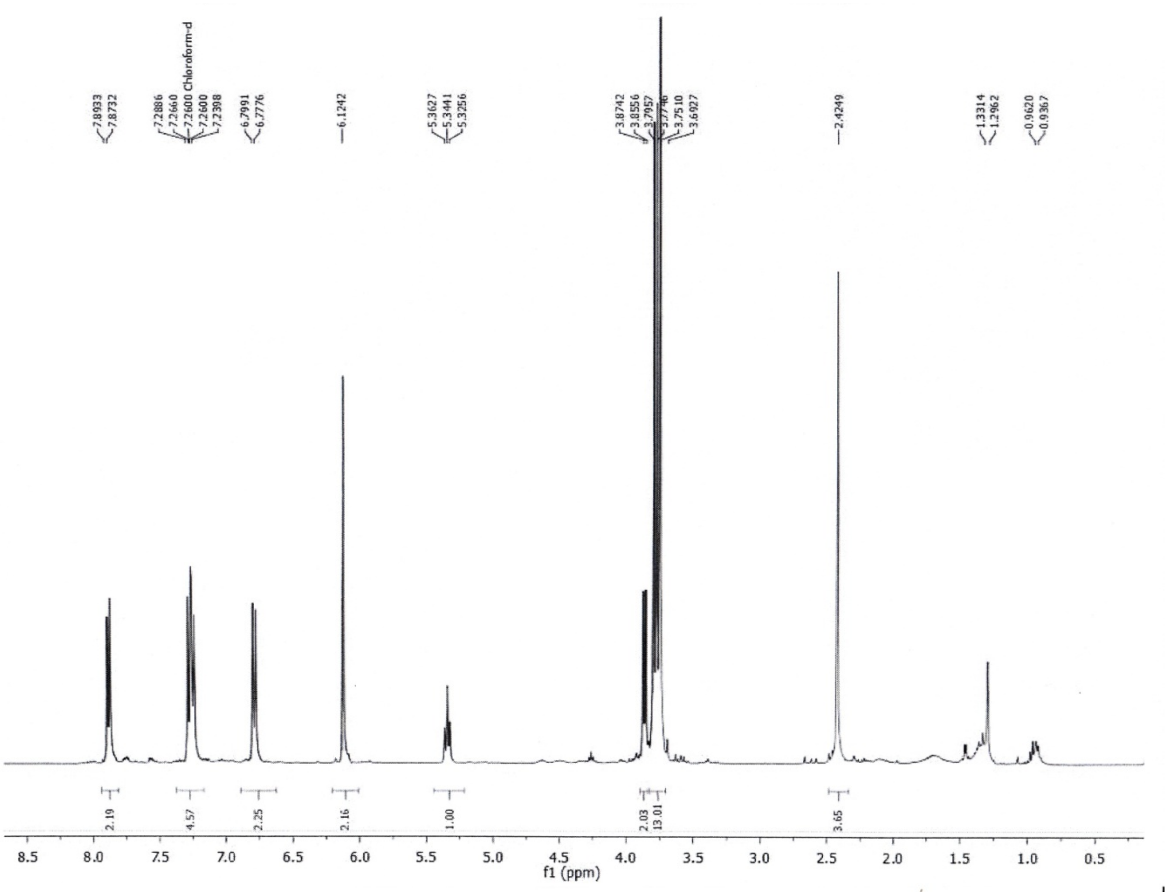

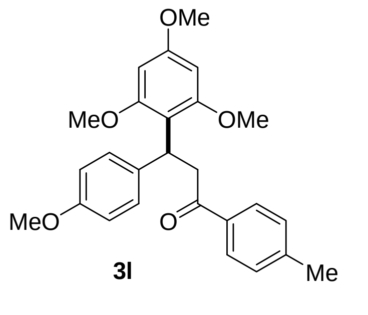

3l

^{13}C-NMR of compound **3l**

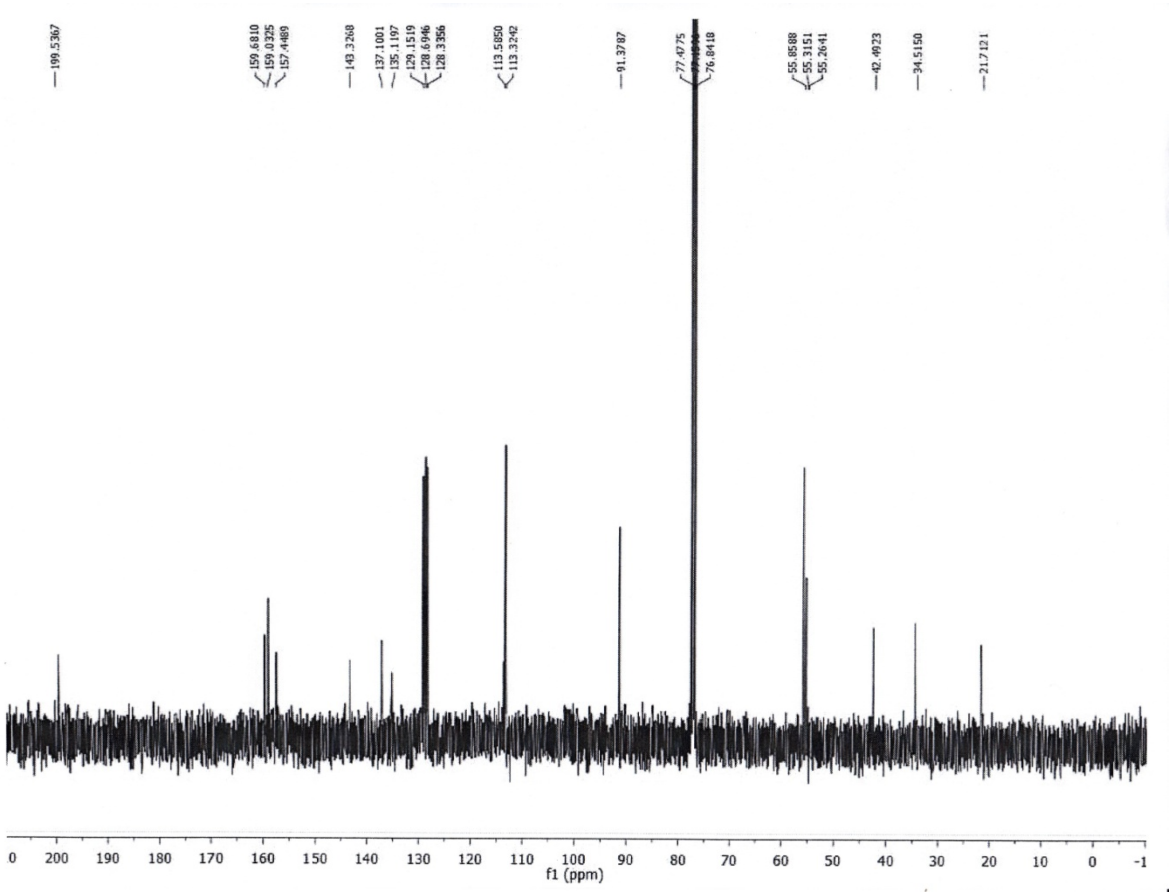

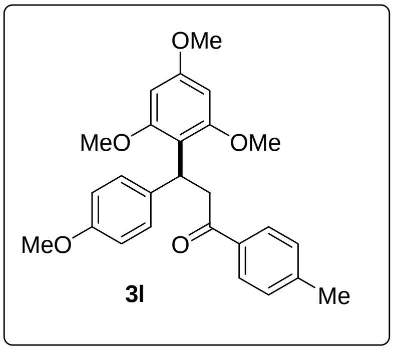

^{1}H-NMR of compound **3m**

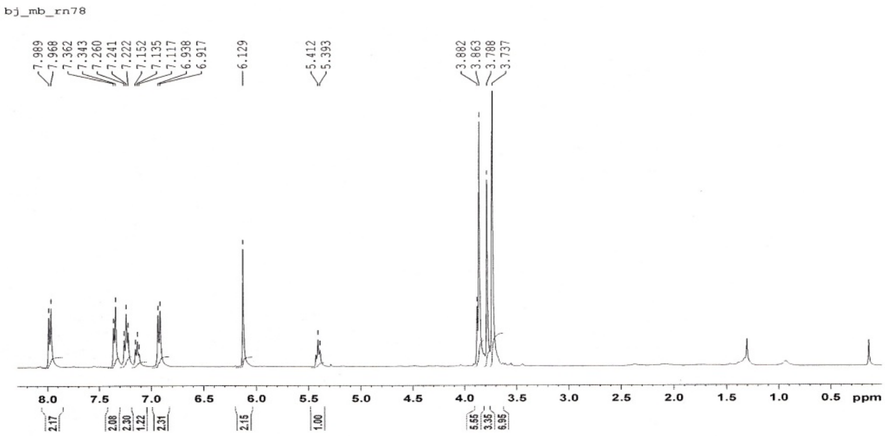

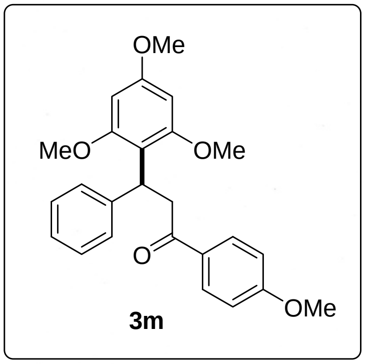

^{13}C-NMR of compound **3m**

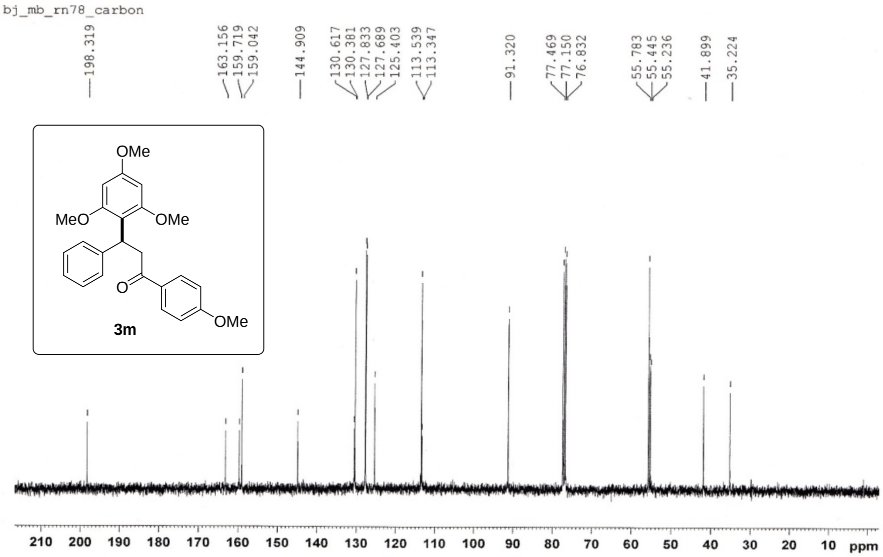

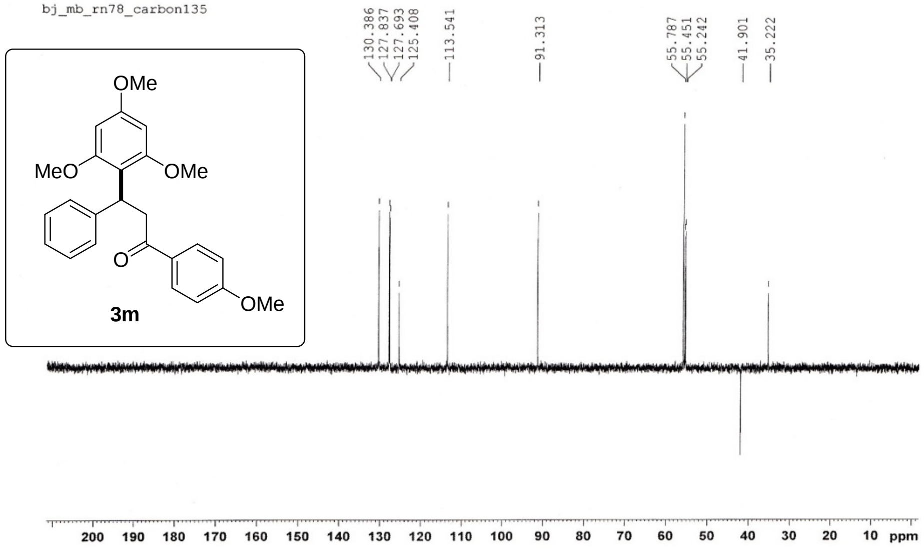

Copy of ^{1}H NMR and ^{13}C Spectra of Friedel-Crafts Alkylated Products 5a-5d

^{1}H-NMR of compound **5a**

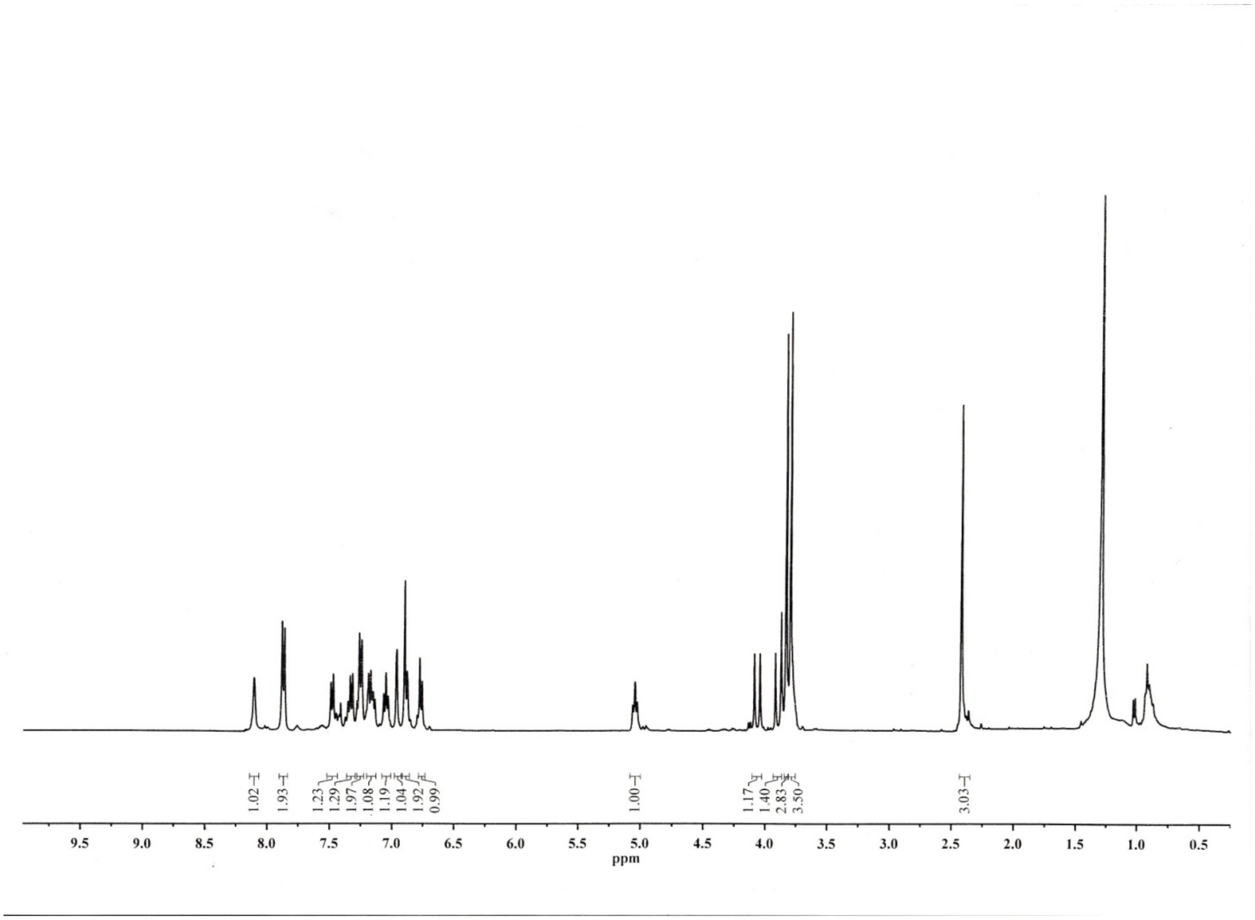

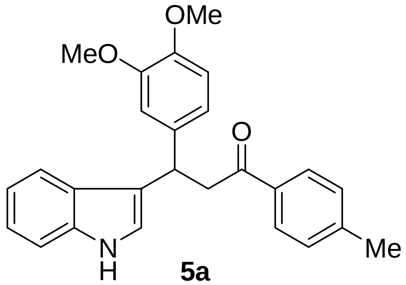

5a

^{13}C-NMR of compound **5a**

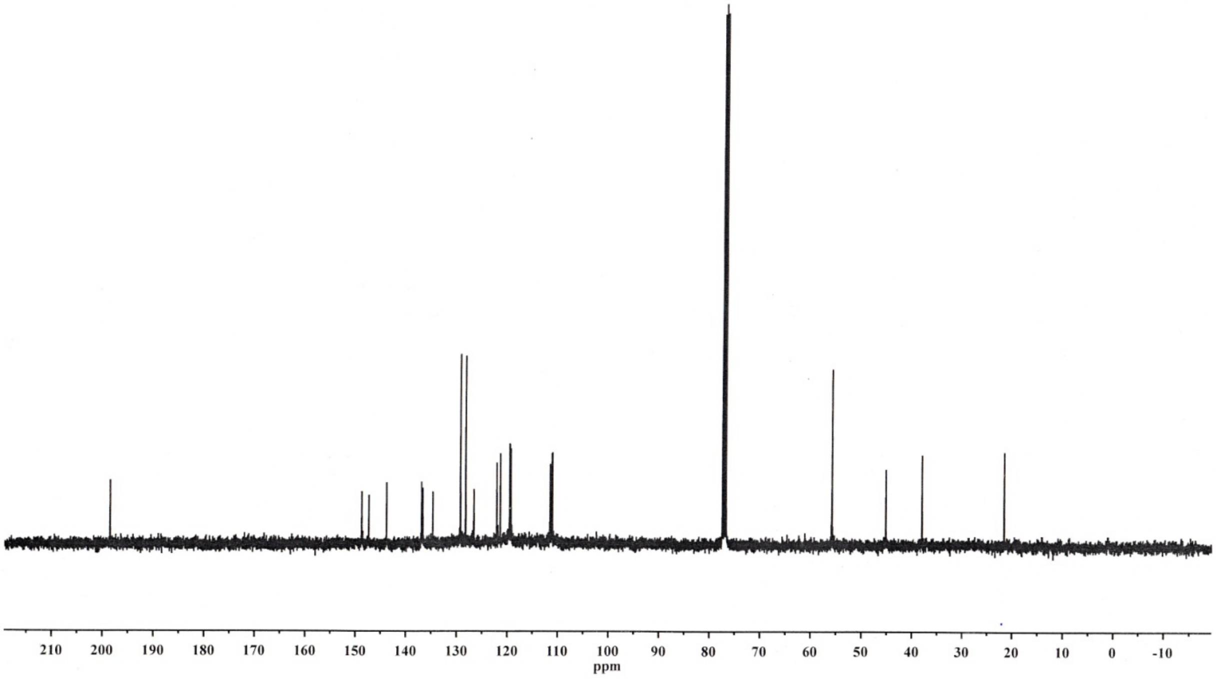

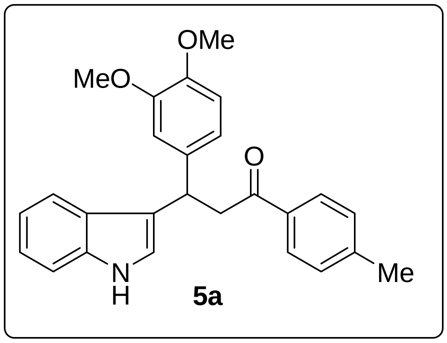

^{1}H-NMR of compound **5b**

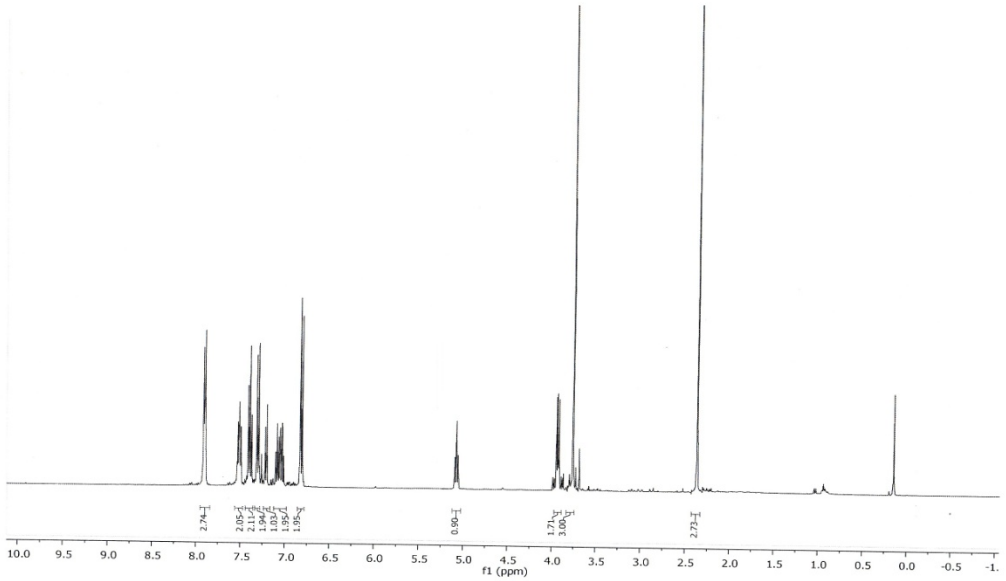

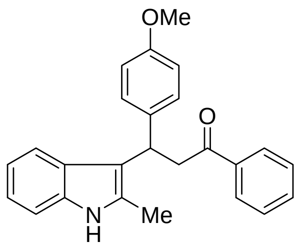

5b

^{13}C-NMR of compound **5b**

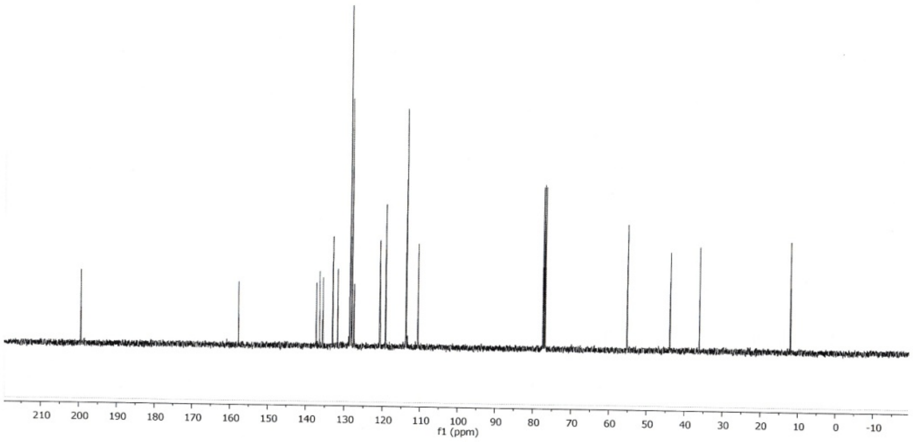

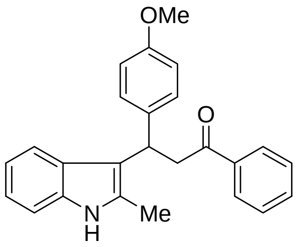

5b

^{1}H-NMR of compound **5c**

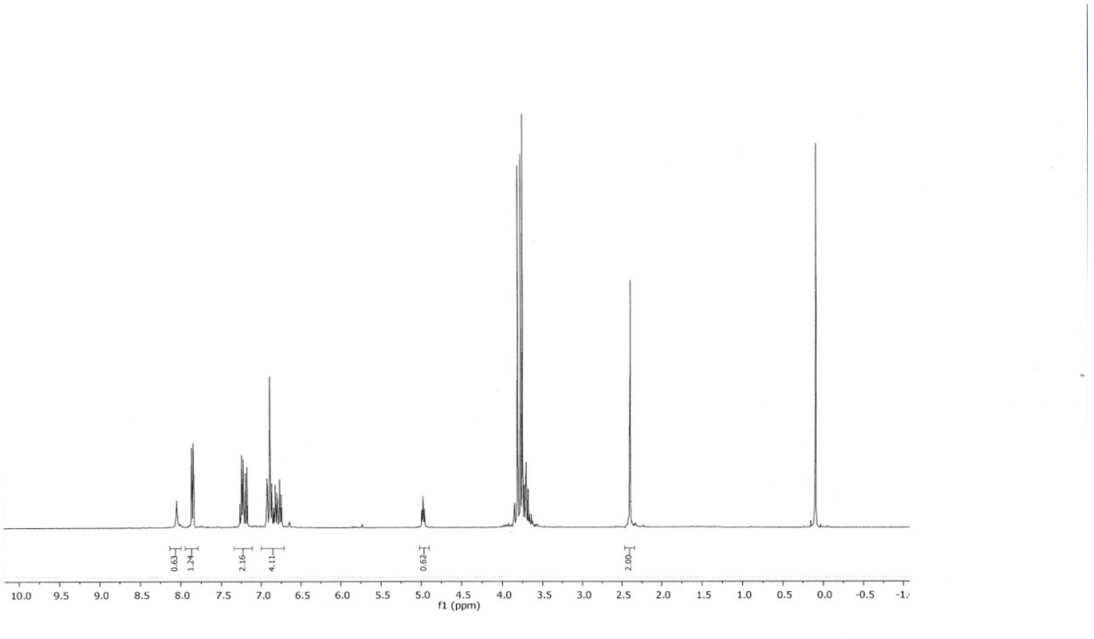

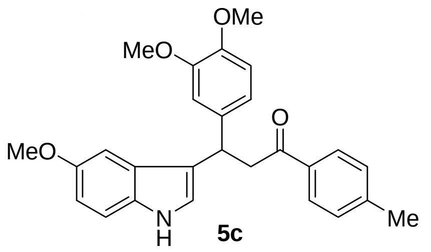

5c

^{13}C-NMR of compound **5c**

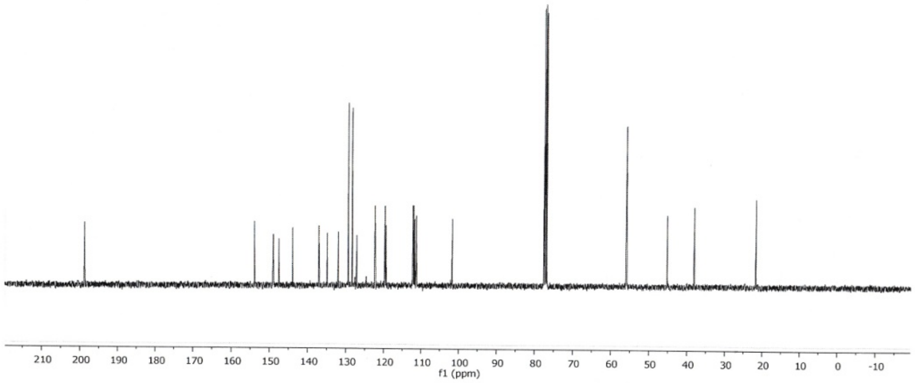

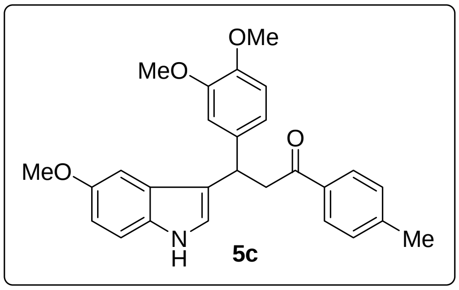

^{1}H-NMR of compound **5d**

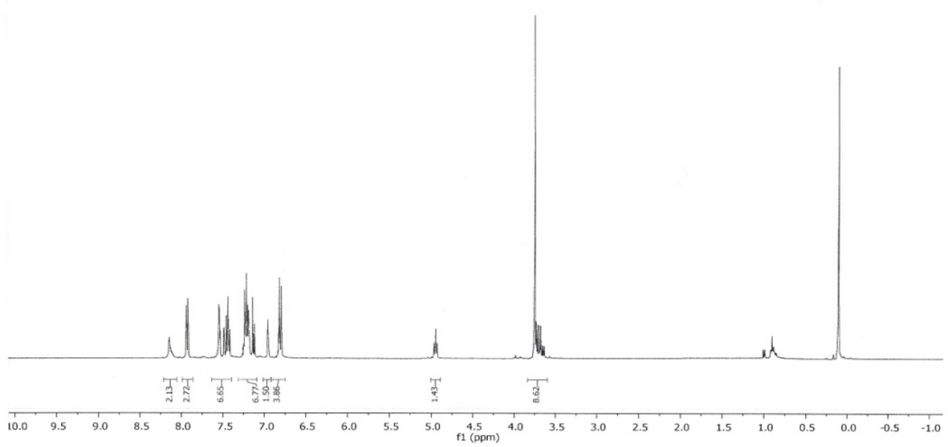

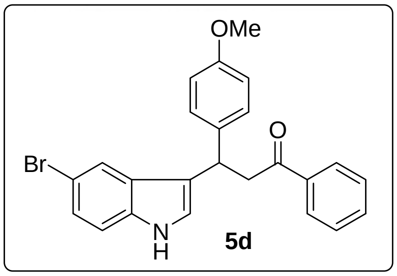

^{13}C-NMR of compound **5d**

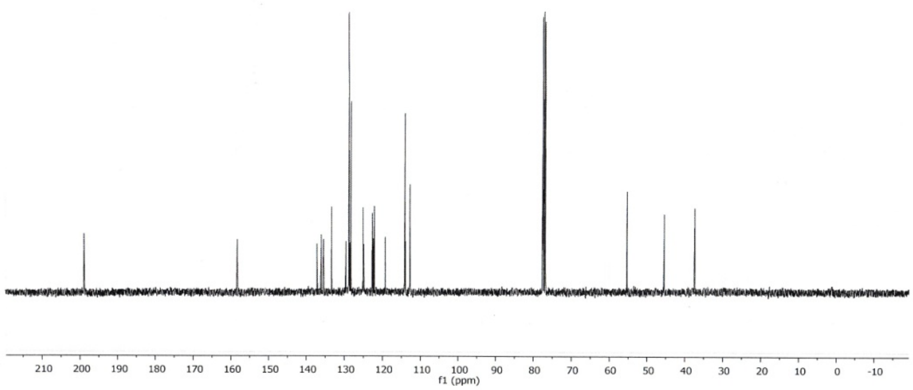

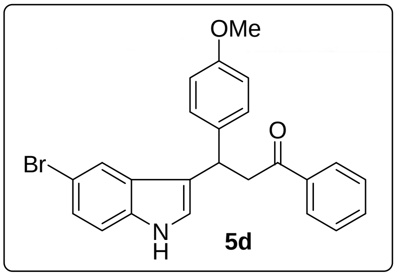

Mechanism Investigation

Scheme 1

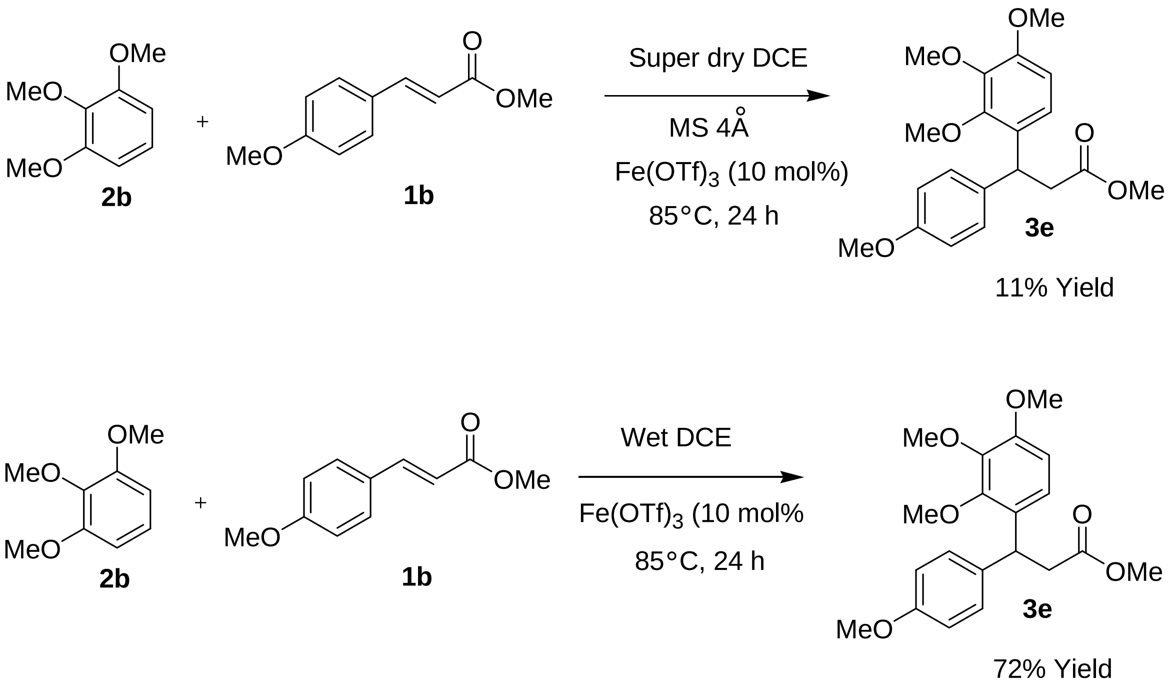

Scheme 2

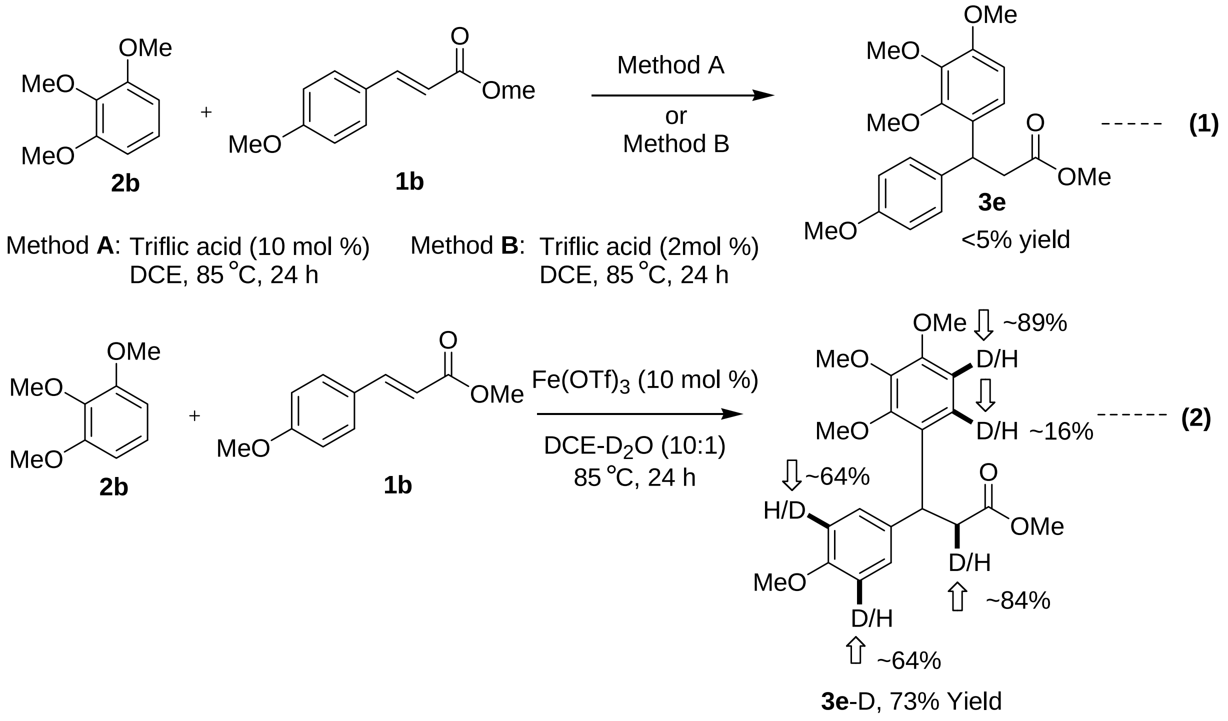

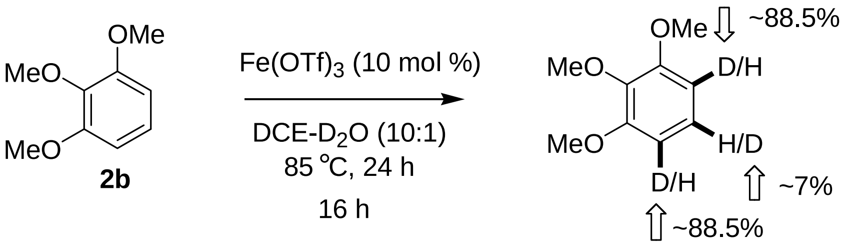

(3)

2b-D/H; quantative Yield

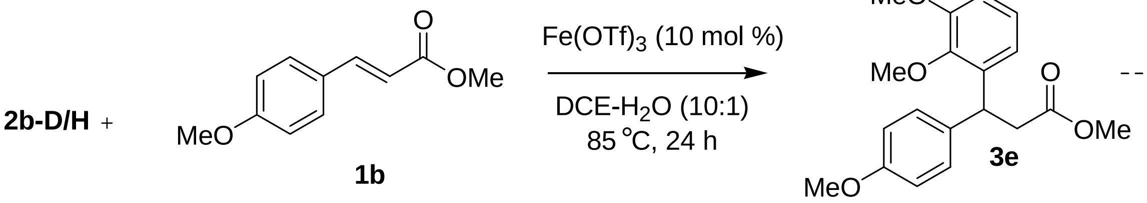

(4)

0% D-incorporation
71% yield

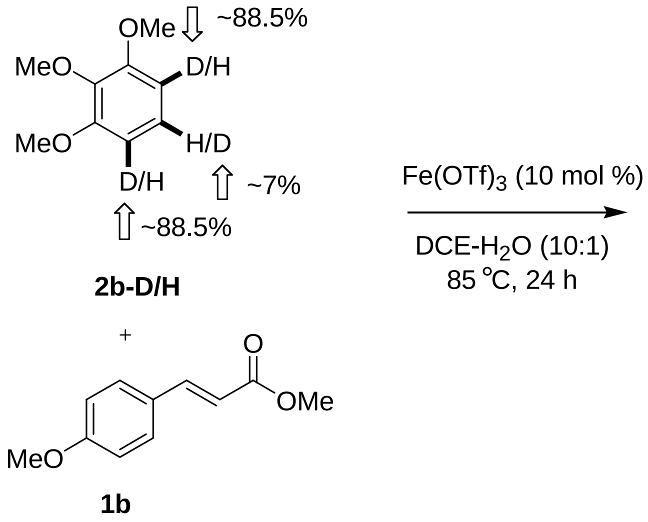

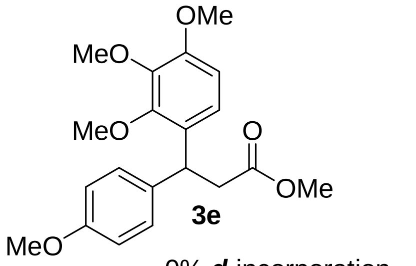

0% ***d***-incorporation
71% yield

$C_{20}H_{24}NaO_6$
Exact Mass: 383.1471
Mol. Wt.: 383.3907
m/e: 383.1471 (100.0%), 384.1504 (22.2%),
385.1538 (2.4%), 385.1513 (1.2%)

Spectral data of 3e-D and 2b-D/H

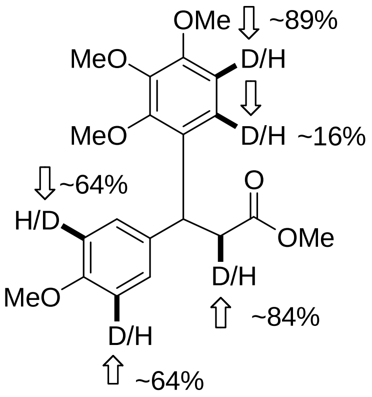

3e-D, 73% Yield

^{1}H NMR (CDCl$_3$, 400 MHz): δ 7.15 (s, 2H), 6.87 (s, 0.84H), 6.80 (d, *J* = 8.0 Hz, 0.71H), 6.62 (d, *J* = 8.0 Hz, 0.11H), 4.77 (d, *J* = 8.0 Hz, 2H), 3.83 (s, 3H), 3.82 (s, 3H), 3.75 (s, 3H), 3.67 (s, 3H), 3.58 (s, 3H), 2.97 (m, 0.84H).

HRMS (EI) calcd for $C_{20}H_{21}D_3O_6$, 386.1656 *m/z* $(M+Na)^+$; Found, 386.1639 *m/z*.

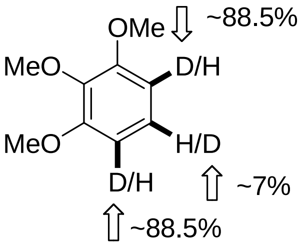

2b-D/H

^{1}H NMR (CDCl$_3$, 400 MHz): δ 6.99 (s, 0.93H), 6.58 (d, *J* = 8.0 Hz, 0.23H), 3.86 (s, 6H), 3.85 (s, 3H).

Copy of ^{1}H NMR and HRMS Spectra with *d*-incorporation in Friedel-Crafts Alkylated Products 3e-D & 2b-D/H

^{1}H-NMR of compound **3e**-D (400 MHz)

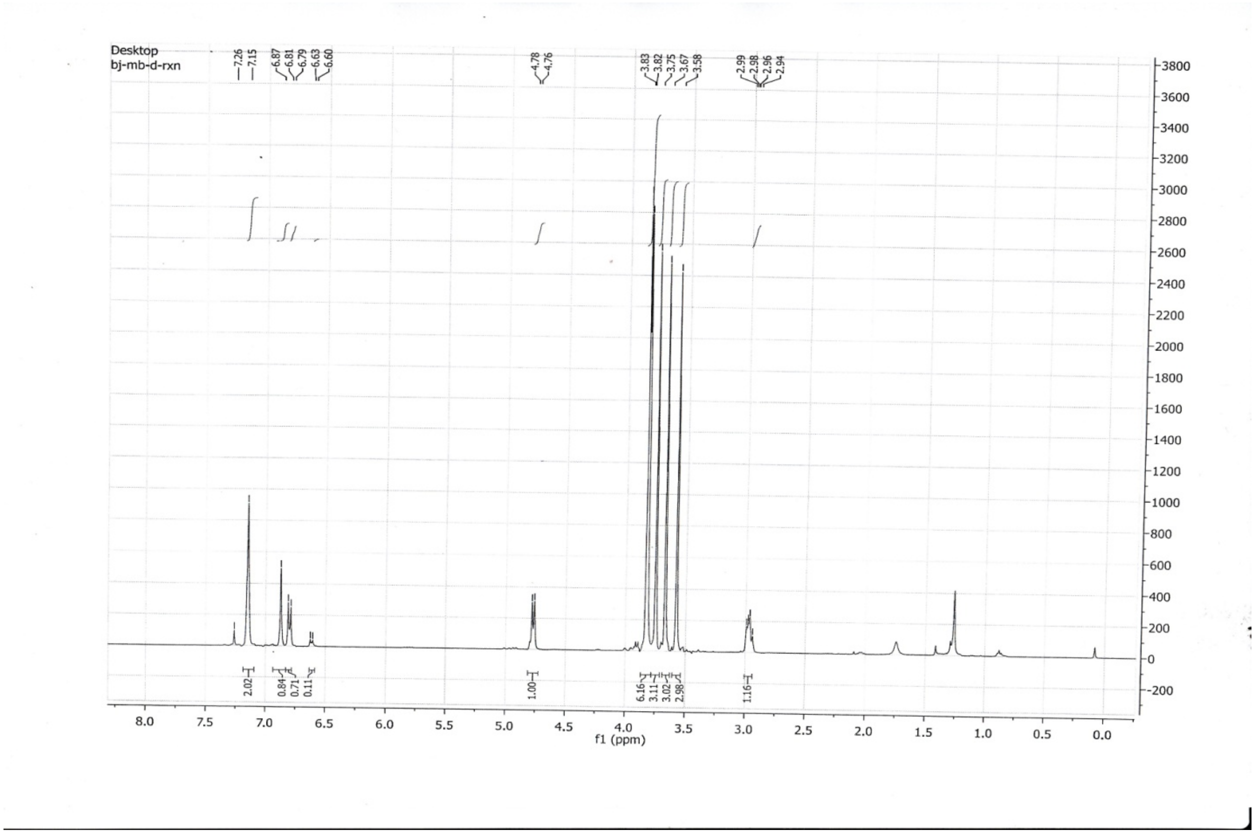

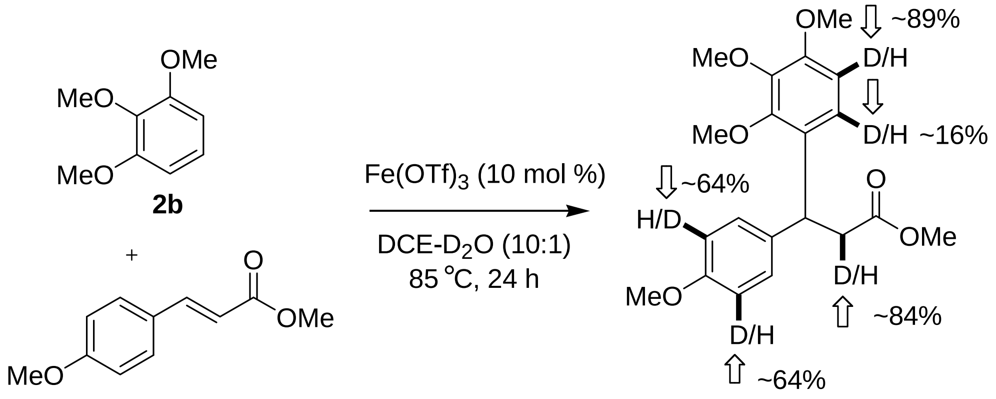

3e-D, 73% Yield

$C_{20}H_{21}D_3NaO_6$
Exact Mass: 386.1656
Mol. Wt.: 386.4089
m/e: 386.1656 (100.0%), 387.1689 (22.2%), 388.1723 (2.4%), 388.1698 (1.2%)

HRMS Spectrum of **3e**-D

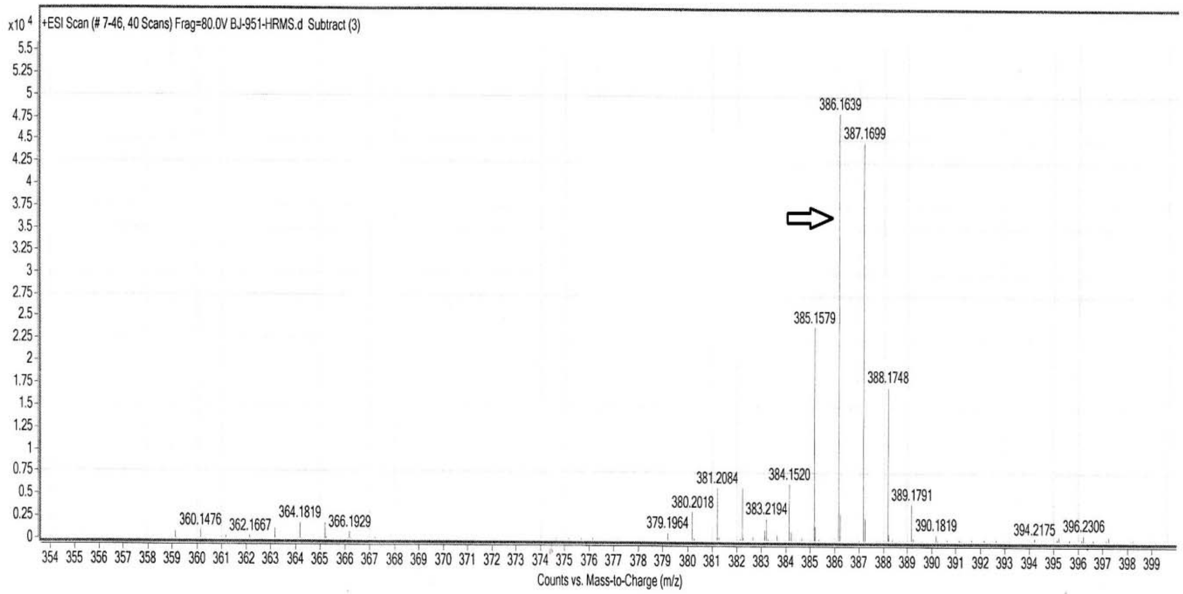

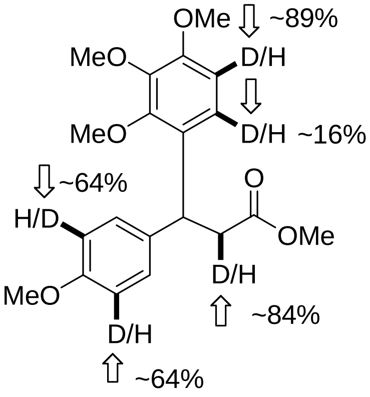

3e-D, 73% Yield

$C_{20}H_{21}D_3NaO_6$
Exact Mass: 386.1656
Mol. Wt.: 386.4089
m/e: 386.1656 (100.0%), 387.1689 (22.2%), 388.1723 (2.4%), 388.1698 (1.2%)

^{1}H-NMR of compound **2b**-D/H (400 MHz)

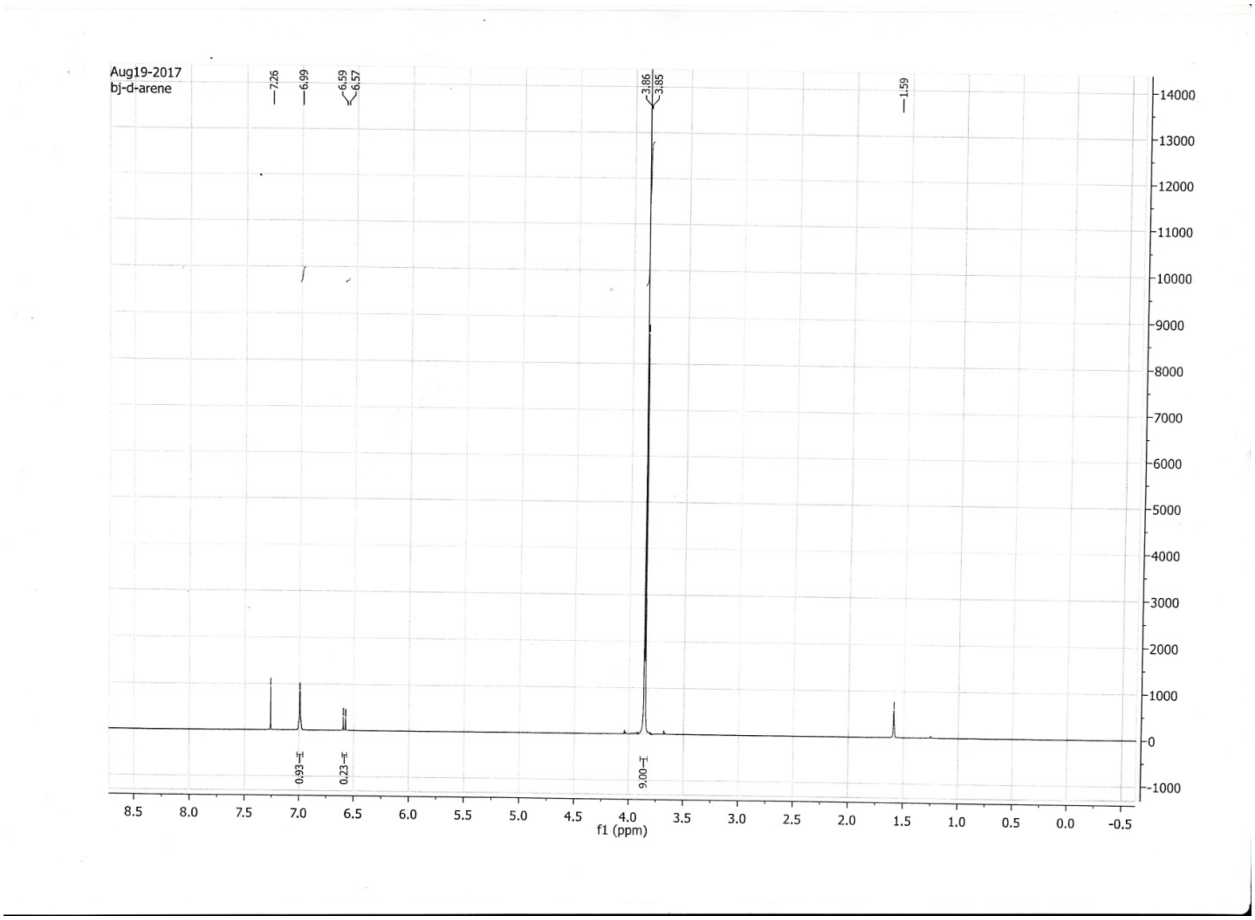

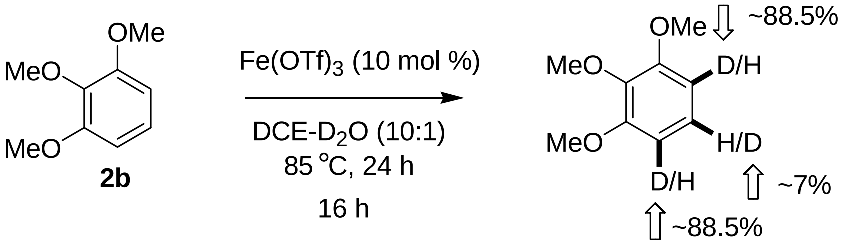

2b-D/H; quantative Yield

^{1}H-NMR of compound **3e** after reaction between **2b**-D/H and alkene **1b** in the presence of DCE-H_2O (10:1) (400 MHz)

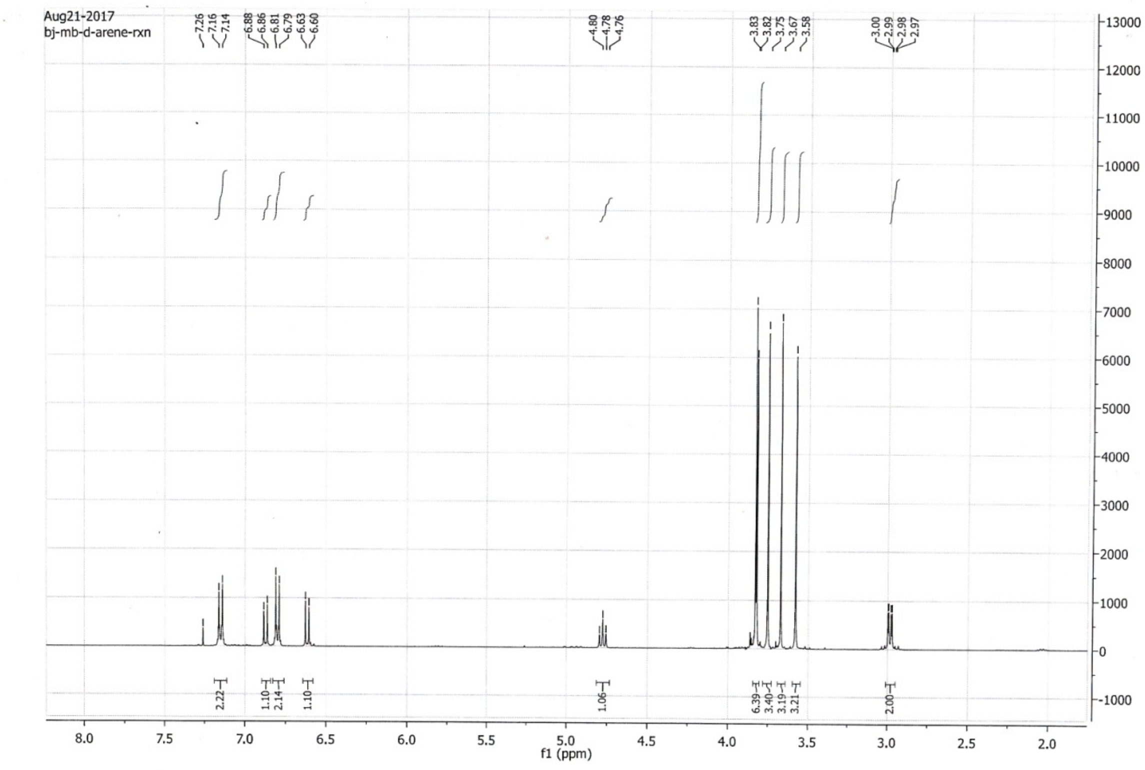

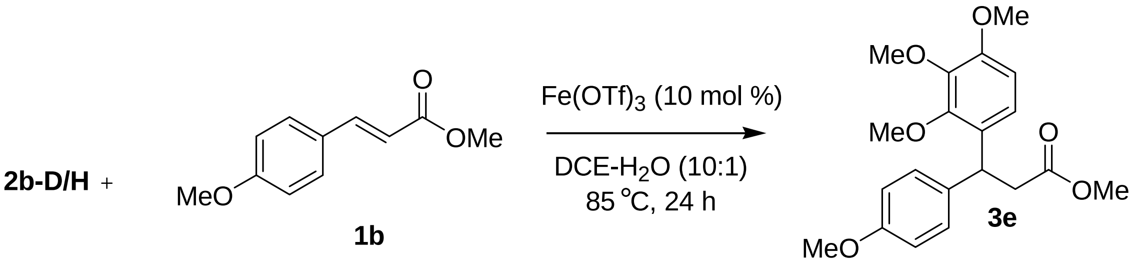

0% D-incorporation
71% yield

HRMS Spectrum of **3e** after reaction between **2b**-D/H and alkene **1b** in the presence of DCE-H_2O (10:1)

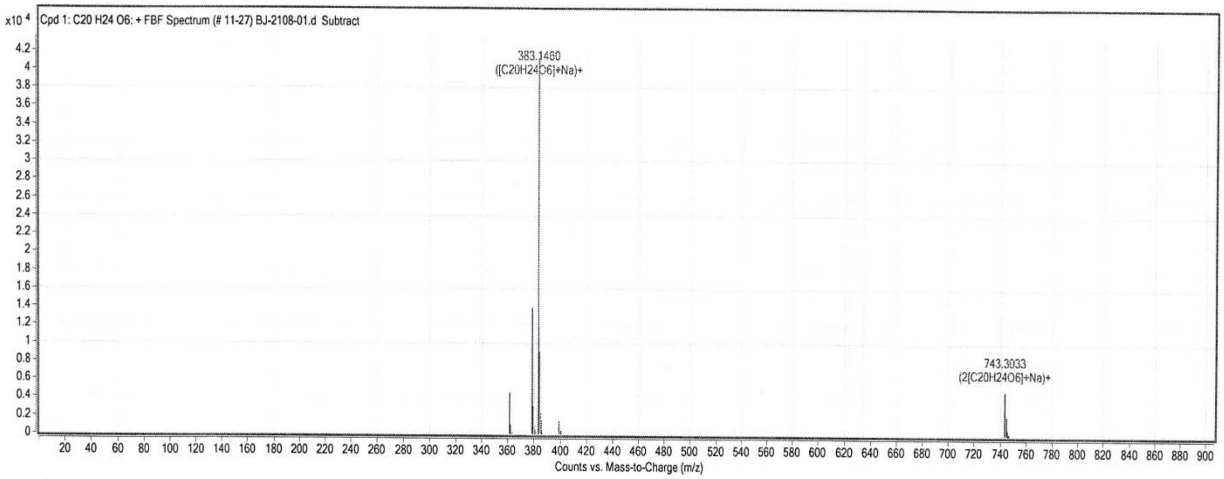

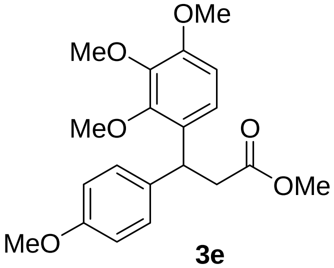

3e

$C_{20}H_{24}NaO_6$
Exact Mass: 383.1471
Mol. Wt.: 383.3907
m/e: 383.1471 (100.0%), 384.1504 (22.2%), 385.1538 (2.4%), 385.1513 (1.2%)
